# The Role of Plasma Membrane Sodium/Hydrogen Exchangers in Gastrointestinal Functions: Proliferation and Differentiation, Fluid/Electrolyte Transport and Barrier Integrity

**DOI:** 10.3389/fphys.2022.899286

**Published:** 2022-05-18

**Authors:** Katerina Nikolovska, Ursula E. Seidler, Christian Stock

**Affiliations:** Department of Gastroenterology, Hannover Medical School, Hannover, Germany

**Keywords:** constipation, cystic fibrosis, diarrhea, differentiation, gastrointestinal tract, NHE, NHE inhibitors, sodium/hydrogen exchange

## Abstract

The five plasma membrane Na^+^/H^+^ exchanger (NHE) isoforms in the gastrointestinal tract are characterized by distinct cellular localization, tissue distribution, inhibitor sensitivities, and physiological regulation. NHE1 (Slc9a1) is ubiquitously expressed along the gastrointestinal tract in the basolateral membrane of enterocytes, but so far, an exclusive role for NHE1 in enterocyte physiology has remained elusive. NHE2 (Slc9a2) and NHE8 (Slc9a8) are apically expressed isoforms with ubiquitous distribution along the colonic crypt axis. They are involved in pH_i_ regulation of intestinal epithelial cells. Combined use of a knockout mouse model, intestinal organoid technology, and specific inhibitors revealed previously unrecognized actions of NHE2 and NHE8 in enterocyte proliferation and differentiation. NHE3 (Slc9a3), expressed in the apical membrane of differentiated intestinal epithelial cells, functions as the predominant nutrient-independent Na^+^ absorptive mechanism in the gut. The new selective NHE3 inhibitor (Tenapanor) allowed discovery of novel pathophysiological and drug-targetable NHE3 functions in cystic-fibrosis associated intestinal obstructions. NHE4, expressed in the basolateral membrane of parietal cells, is essential for parietal cell integrity and acid secretory function, through its role in cell volume regulation. This review focuses on the expression, regulation and activity of the five plasma membrane Na^+^/H^+^ exchangers in the gastrointestinal tract, emphasizing their role in maintaining intestinal homeostasis, or their impact on disease pathogenesis. We point to major open questions in identifying NHE interacting partners in central cellular pathways and processes and the necessity of determining their physiological role in a system where their endogenous expression/activity is maintained, such as organoids derived from different parts of the gastrointestinal tract.

## Introduction

Na^+^/H^+^ exchangers (NHEs) belong to the solute carrier (SLC) nine family and represent one group out of the most evolutionary conserved transport proteins, since the basic Na^+^/H^+^ exchange mechanism is conserved and identified among different species from procaryotes to mammals. The NHEs exchange intracellular H^+^ ions for extracellular Na^+^ in 1:1 stoichiometry. This process is used for transepithelial Na^+^ transport and water absorption, and for intracellular pH (pH_i_) regulation, which is essential for cell function and survival. In the gastrointestinal tract, eight NHE isoforms (with the exception of NHE5) have been identified and found to be localized intracellularly (NHE6,7 and 9) or in the plasma membrane (NHE1,2,3, 4, and 8). The plasma membrane NHEs can be sub-located to the basolateral (NHE1 and 4) or apical (NHE2,3 and 8) membrane of a polarized gastrointestinal epithelial cell. In the last 30 years since their discovery, NHEs have been extensively investigated and the findings have been summarized in a number of excellent reviews (Orlowski and Grinstein, 2004; Zachos et al., 2005; Kiela et al., 2006; Donowitz et al., 2013; Gurney et al., 2017; Xu et al., 2018; Cao et al., 2019; Pedersen and Counillon, 2019). Yet, new results reveal novel aspects of their physiological and pathophysiological role and make them still very interesting for scientists. This review will focus on the plasma membrane-located NHEs in the gastrointestinal tract by: 1) summarizing and discussing original studies, starting from identification, cloning and characterization of the NHEs to the newest state of the art studies describing their function; 2) describing each plasma membrane NHE isoform with their different modes of transcriptional/translational regulation, tissue distribution, and physiological and pathophysiological roles; and 3) indicating still unanswered questions, debates, and challenges in deciphering the role of gastrointestinal NHEs.

## NHE1 in the Gastrointestinal Tract

### Early Studies Explored the Mechanisms of pH_i_-Maintenance in Gastroduodenal Epithelial Cells

In the gastroduodenal epithelia, the mechanisms of pH_i_-regulation were explored very early, because of the interest in how these epithelia withstand the very high luminal proton concentration. Far before the molecular identification of the different NHE isoforms, basolateral amiloride-analogue sensitive Na^+^/H^+^ exchange has been functionally identified in the different cell types of the gastric epithelium (Paradiso et al., 1987; Seidler et al., 1989), in chambered gastric (Horie et al., 1992; Seidler et al., 1995) and duodenal mucosa (Paimela et al., 1992), in basolateral gastric and intestinal membrane vesicles (Zamir et al., 1992; Lamprecht et al., 1993), as well as in an intestinal cell line (Watson et al., 1991) and in esophageal cells (Layden et al., 1990; Tobey et al., 1992).

**FIGURE 1:**
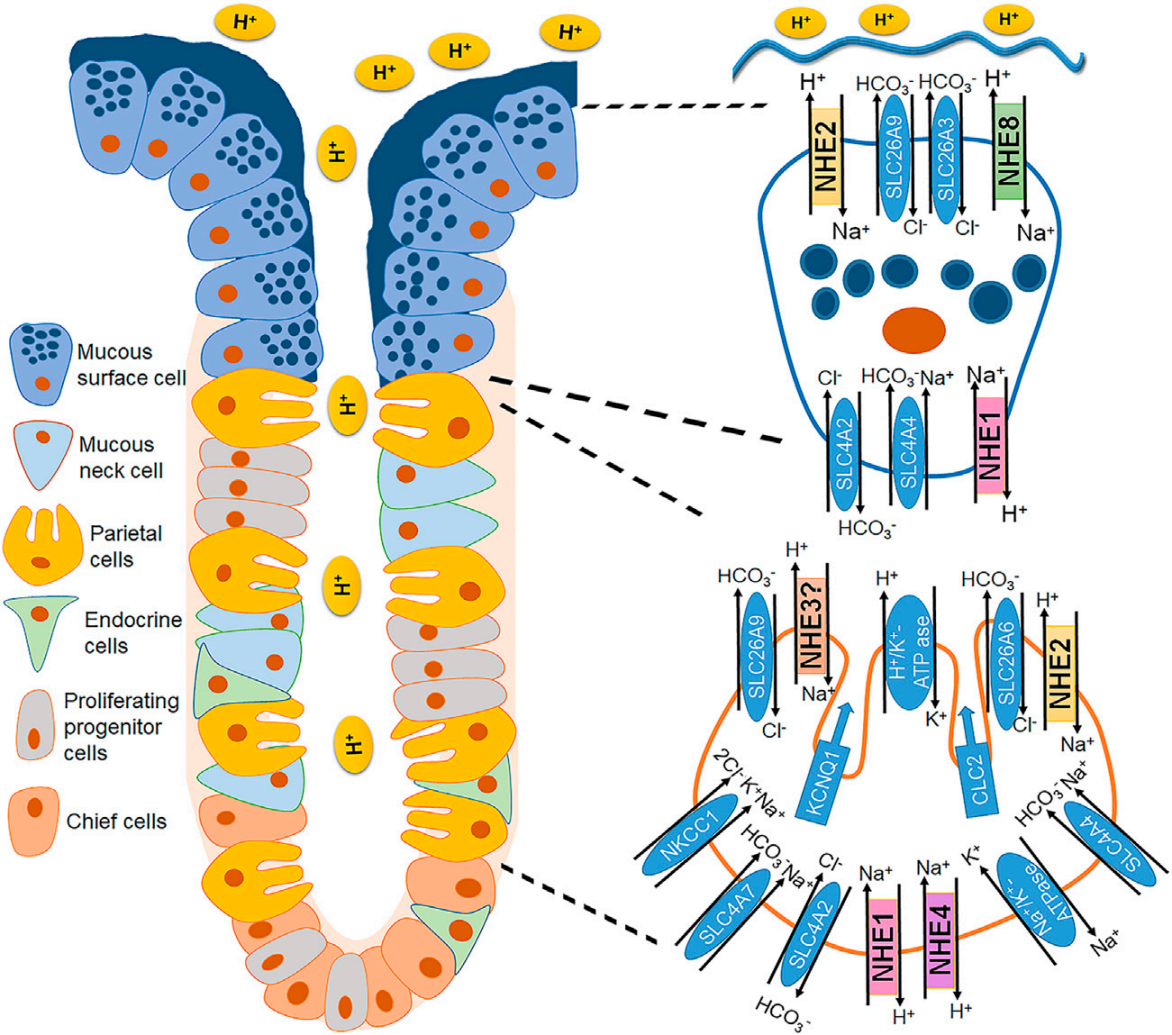
Localization of the different plasma membrane NHE isoforms in the gastric epithelial cells. A corpus gastric gland is schematically represented with focus on a parietal cell (bottom right) and a mucous surface cell (top right), and the NHEs and other ion transporters involved in acid/base homeostasis. NHE1 is found in the basolateral membrane of the surface and neck mucous cells, chief cells, and parietal cells (Stuart-Tilley et al., 1994; Rossmann et al., 2001). NHE2 expression was demonstrated in rabbit gastric mucous, chief and parietal cells (Seidler et al., 1997; Rossmann et al., 2001) and later confirmed by immunohistochemical staining in the apical membranes of gastric surface epithelium (Xue et al., 2011). NHE3 expression has been shown in the rat (Orlowski et al., 1992), human, and guinea pig (Kulaksiz et al., 2001), but not in rabbit gastric mucosa (Rossmann et al., 2001), and functionally identified in the parietal cell apical membrane (Kirchhoff et al., 2003). NHE4 is expressed in the basolateral membrane of parietal and chief cells and to a lesser extent in mucous cells (Pizzonia et al., 1998; Rossmann et al., 2001). NHE8 was described to be apically located in the mouse fundic and pyloric glands (Xu et al., 2013).

Several studies emphasized the involvement of parietal cell Na^+^/H^+^ exchange in orchestrating the various ion transport activities that alter during stimulation of acid secretion, with conflicting results (Muallem et al., 1988; Paradiso et al., 1989; Seidler et al., 1992; Bachmann et al., 1998). After the identification of the NHE subtypes, it became clear that at least three NHE candidates, namely NHE1, NHE2 and NHE4 are strongly expressed in parietal cells (Rossmann et al., 2001) (Figure 1).

A few studies successfully demonstrated an involvement of basolateral Na^+^/H^+^ exchange in the maintenance of a neutral pH in the gastroduodenal epithelial cells during a luminal acid load and uncovered the importance of basolateral/systemic bicarbonate availability to counteract the luminal proton load (Kiviluoto et al., 1990; Kaneko et al., 1992; Kaunitz and Akiba, 2002). While intuitively, NHE1 appears as the ideal candidate to convey this acid-protective function and high NHE1 expression has been later documented in the mucous neck cells neighboring the parietal cells (Stuart-Tilley et al., 1994), functional experimental evidence for this assumption is lacking.

### Early Studies on the Involvement of Na^+^/H^+^ Exchange in Proliferation of Gastrointestinal Cells

In the early eighties of the last century, scientists recognized that: 1) growth factors and other signaling events related to cellular proliferation stimulated Na^+^/H^+^ exchange and resulted in a sustained increase in intracellular pH_i_ in the absence of CO_2_ and HCO_3_^−^ (Pouysségur et al., 1982; Moolenaar et al., 1983; Berridge et al., 1985), 2) an increased activity of Na^+^/H^+^ exchange was a feature of cell proliferation in studied cellular systems including immune cells, malignant cells and epithelial cells (Moolenaar et al., 1981; Mahnensmith and Aronson, 1985; Lagarde and Pouysségur, 1986; Mendoza, 1988), and that 3) pharmacological inhibition of Na^+^/H^+^ exchange interfered with certain essential steps in fibroblast proliferation (Cassel et al., 1987; L'Allemain et al., 1984). In parallel, experimental evidence showed that although an activation of Na^+^/H^+^ exchange did occur in growth factor-induced proliferation, resulting in cytoplasmic alkalization in the absence but not the presence of CO_2_ and HCO_3_^−^, this was not essential for the proliferative response, at least not in immune cells (Mills et al., 1985), in embryonic fibroblasts (Besterman et al., 1984), or in a breast.

In the esophageal epithelium, an increased proliferative rate in the basal cells after experimental exposure of the esophageal mucosa to luminal acid was detected (De Backer et al., 1985; Carpizo et al., 1998). Similar finding was observed in specimens from esophageal mucosa from reflux patients with chronic acid reflux but without inflammation (Ismail-Beigi et al., 1970). An interesting event during exposure of esophageal epithelial cells to a low extracellular pH was an increase in Na^+^/H^+^ exchange rates (Layden et al., 1992; Tobey et al., 1992). A study suggested a causal relationship between the elevated proliferation and NHE activity in the esophageal mucosa and showed that NHE1 (which was by then molecularly identified) was the only NHE isoform expressed in esophageal mucosa (Shallat et al., 1995). The authors pointed out that repeated episodes of luminal acidification might, *via* increased NHE1-mediated Na^+^/H^+^ rates that primarily serve as protective pH_i_-regulators, have negative consequences by sustaining epithelial proliferation and favoring malignant growth. This concept was further experimentally validated (Fitzgerald et al., 1996; Fitzgerald et al., 1998). Thus, the topic of both a beneficial, protective action of Na^+^/H^+^ exchange by NHE1, as well as of a negative role of NHE1 hyperactivity favoring malignant growth was discussed early in gastrointestinal epithelia.

Over the following decades, the mechanisms of an altered pH_i_ climate in many cancers including esophageal, gastric and colonic cancer, the consequences for malignant proliferation, as well as the importance of the NHE1 isoform in the maintenance of malignant growth and the potential to influence malignant growth by manipulating NHE1 activity, was intensely studied, as well as reviewed (Reshkin et al., 2000; Fang et al., 2008; Liu et al., 2008; Boedtkjer et al., 2012; Sharma et al., 2015; Parks et al., 2017; Stock and Pedersen, 2017; Cao et al., 2019; Cardone et al., 2019; Hu et al., 2021). Because of the existence of many excellent reviews, the present one will not include the role of NHE1 in gastrointestinal malignancies.

### Role of NHE1 in Nonmalignant Gastrointestinal Epithelia

**FIGURE 2:**
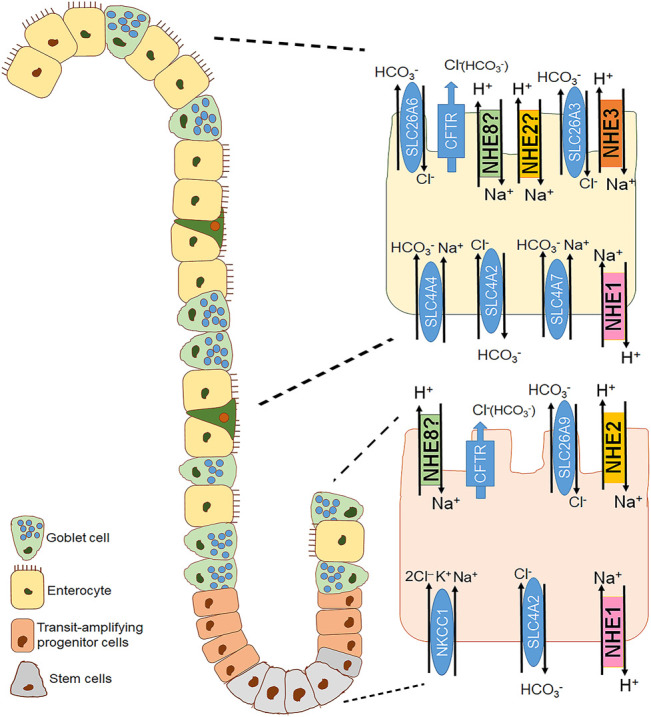
Distribution of the different plasma membrane NHE isoforms along the crypt villus axis of the small intestine. A crypt villus axis of the small intestine with a focus on the crypt (bottom right) and villus cell (top right) is schematically represented. NHE1 is the house keeping NHE isoform located in the basolateral membrane of both cryptal and villus cells of the small intestine (Bookstein et al., 1994a; Dudeja et al., 1996). NHE2 is found in the apical membrane of the intestinal epithelial cells, presumably more in the cryptal compared to the villus region (Hoogerwerf et al., 1996; Bookstein et al., 1997). NHE3 is the major brush border NHE isoform responsible for Na^+^ absorption and located in the apical membrane of the intestinal surface cells (Bookstein et al., 1994a; Grant et al., 2015; Foulke-Abel et al., 2016). NHE8 is also expressed in the apical membrane of the epithelial cells in the small intestine, proposedly along the crypt villus axis (Xu et al., 2005).

**FIGURE 3:**
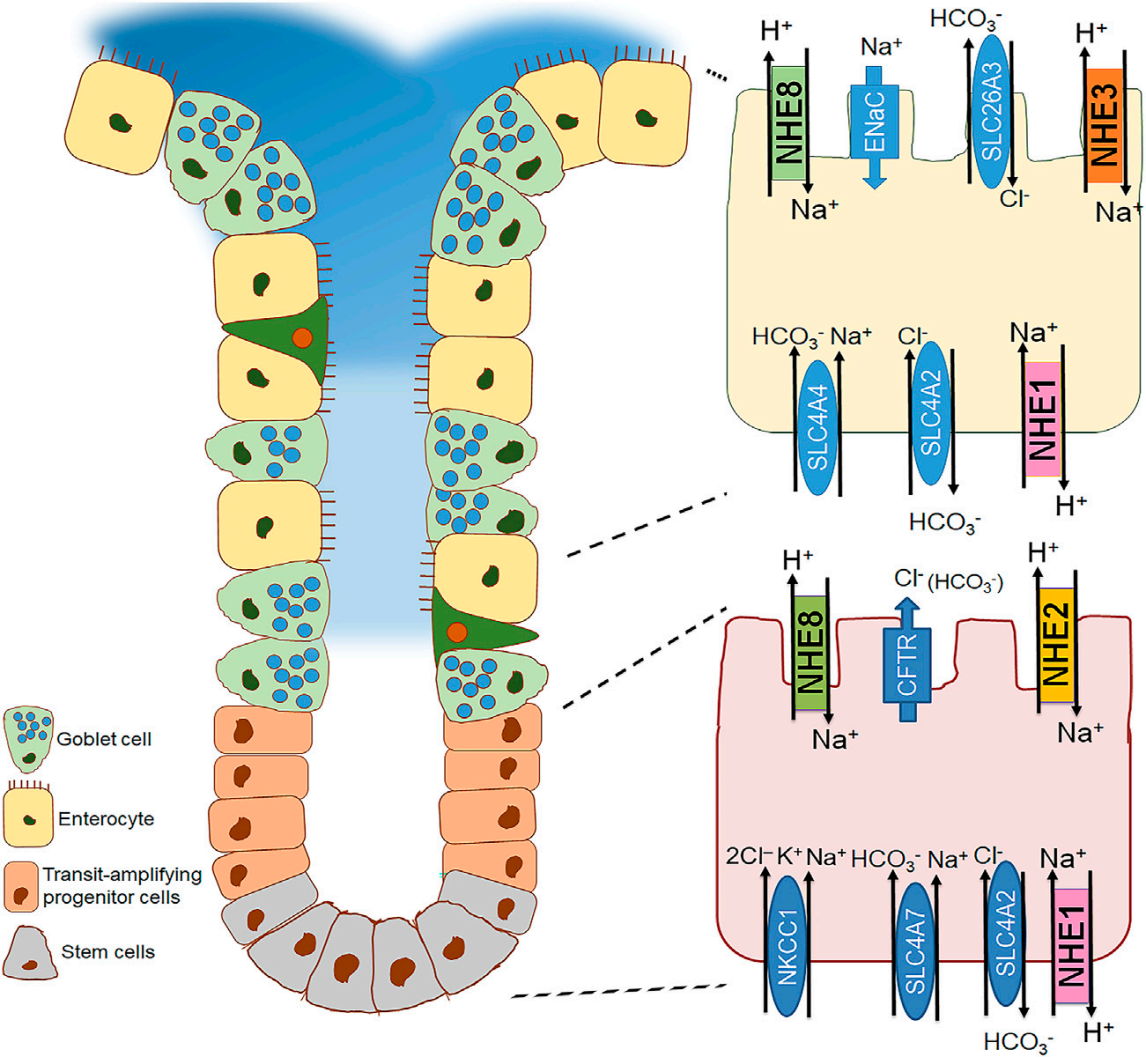
Distribution of the different plasma membrane NHE isoforms along the colonic cryptal axis in the mouse colon. Crypt of the small intestine with a focus on the basal (bottom right) and surface cell (top right) is schematically represented. NHE1 is expressed along the cryptal axis in the basolateral membrane of the epithelial cells. NHE2 is expressed in the apical membrane predominantly in the cryptal region (Chu et al., 2002; Bachmann et al., 2004; Guan et al., 2006). In contrast, NHE3 is found in the apical membrane of the surface colonic epithelial cells (Bachmann et al., 2004; Guan et al., 2006; Talbot and Lytle, 2010; Nikolovska et al., 2022). NHE8 is expressed ubiquitously along the cryptal axis in the apical membrane of colonocytes (Xu et al., 2019).

The NHE1 isoform is believed to be ubiquitously expressed (although this was of course not documented in all cell types), and localized to the basolateral membrane of epithelial cells. An expression of the NHE1 isoform was documented in the esophagus (Shallat et al., 1995), in the different gastric epithelial cells (Rossmann et al., 2001; Stuart-Tilley et al., 1994; Xu et al., 2008), the salivary glands (He et al., 1997; Robertson et al., 1997), the small and large intestine (Bookstein et al., 1994a; Dudeja et al., 1996), the gallbladder (Narins et al., 2004), the biliary ducts (Spirlì et al., 1998) and the pancreas (Roussa et al., 2001) of different species (Figures 1–3). Anti-NHE1 isoform specific antibodies were used for localization, but yielded conflicting results, because specificity of an antibody can only be tested with a high confidence level in knockout mice for the respective antigen. Since in most of these organs, several NHEs are expressed, the functional relevance of NHE1 in these organs requires further study. Based on studies in expression systems, in fibroblasts, embryonic and tumor cell lines, as well as in native epithelial cells, NHE1 is considered to perform “housekeeping” functions, such as pH_i_ and cell volume maintenance, and to be involved in cell proliferation and migration (reviewed in: (Counillon and Pouysségur, 1993; Noël and Pouysségur, 1995; Orlowski and Grinstein, 2004; Wakabayashi et al., 1992). NHE1 was also found to establish important protein-protein interactions, which expands the role of NHE1 beyond its transport function (reviewed in: (Baumgartner et al., 2004; Parker et al., 2015; Pedersen, 2006). Since the inhibitory potential of the “NHE1-selective” inhibitors is also very high for NHE2 and NHE8 (not tested for the intracellular isoforms NHE6, 7 and 9), a selective inhibition of NHE1 by amiloride derivatives or by the “NHE1-selective” inhibitors HOE694 or HOE642 is not possible *in vivo* (Table 1). However, these substances were useful to functionally localize the different NHEs to the apical vs. basolateral membranes in the Caco2BBe intestinal epithelial cell line (Yu et al., 2019; Zhou et al., 2021). In the absence of external CO_2_ and HCO_3_^−^, the NHE1 isoform mediates a very rapid pH_i_-recovery after an experimental intracellular acid load, evidenced by the virtually total inhibition of pH_i_ recovery by 3 µM HOE642 in the basolateral bath solution of a perfusion chamber that can separately perfuse the apical and the basolateral membrane. In the presence of CO_2_ and HCO_3_^−^ in the basolateral perfusate, Na^+^,HCO_3_^−^ cotransporters are also able to normalize pH_i_ after an acid load, which is consistent with the high expression levels of the electrogenic Na^+^,HCO_3_^−^ cotransporter NBCe1 (SLC4A4) and in particular the electroneutral Na^+^,HCO_3_^−^ cotransporter NBCn1 (SLC4A7) in Caco2BBe cells (and in native enterocytes). However, when an NHE1-selective concentration of HOE642 is added to the basolateral perfusate of fully differentiated filter-grown Caco2BBe cells cultured in a HCO_3_^−^ containing culture medium, pH_i_ does not change, because the highly pH_i_-dependent NHE1 is quiescent at the high resting pH_i_ of Caco2BBe cells (Wakabayashi et al., 2003). Whether or not NHE1 is quiescent at resting pH_i_ in native gastric and intestinal epithelial cells is unknown, but pancreatic acinar cells have been found to critically depend on NHE1 for pH_i_ maintenance both in the presence and absence of CO_2_ and HCO_3_^−^ (Brown et al., 2003).

**TABLE 1:** Sensitivity of the gastrointestinal NHE isoforms to the most commonly used drugs.

	Inhibitory Potency (IC50 [µM])					
**Inhibitor**	**NHE1**	**NHE2**	**NHE3**	**NHE4**	**NHE8**	**Comment**
amiloride	5.3	1.4	>100	813		inhibits also ENaC, T-type Ca^2+^ channels (Dai et al., 2007), and uPAR (Xu et al., 2015b)
benzamil	0.08	320				inhibits also ENaC, NCX (Teiwes and Toto, 2007), and TRPP3 (Dai et al., 2007)
cimetidine	51	330	1,000			
clonidine	210	42	620			
DMA	0.023	0.250	14			500 µM inhibits NHE4 (Bookstein et al., 1994b)
EIPA	0.02	0.079	3.3	>10	<1 (Wiebe et al., 2019)	Inhibits TRPP3 at 10.5 µM (Dai et al., 2007)
harmaline	140	330	1,000			
HOE642	0.05	3	1,000		<3	exact IC_50_ for NHE8 not known
HOE694	0.085		640		<10	exact IC_50_ for NHE8 not known
S3226	3.6	80	0.02		<80 (Xu et al., 2008)	
tenapanor			0.005–0.010			highly selective

The table summarizes values provided in the main text and in a chapter by Kiela and Ghishan (2006).

The generation of knockout mice for the different epithelial NHE isoforms resulted in unexpected gastrointestinal phenotypes. The NHE1 knockout (*scl9a1* ^−/−^) mouse dies young because of severe epilepsy, but does not show conspicuous gastrointestinal abnormalities (Bell et al., 1999), in contrast to the mice deficient for NHE2, NHE3, NHE4 and NHE8 (Schultheis et al., 1998a; Schultheis et al., 1998b; Gawenis et al., 2005; Xu et al., 2012), which are described later in this review. The gastric epithelium of *nhe1* ^−/−^ mice shows abnormalities in the interstitial space of the gastric glands, but neither the numbers of the different gastric epithelial cells nor the ultrastructural features of the parietal cells were different from those of the wild type (Miller et al., 2002). Given the high expression of NHE1 in all gastric epithelial cell types (Seidler et al., 1997; Rossmann et al., 2001), the normal appearance of *nhe1* ^−/−^ parietal cells was surprising. A dilation of the interstitial spaces was also observed as a feature of NERD (non-erosive esophageal reflux disease) as a sign of acid exposure of the esophageal epithelium (Tobey et al., 1996; Solcia et al., 2000; Caviglia et al., 2005), a finding that receded upon treatment with proton pump inhibitors (Calabrese et al., 2005; Xue et al., 2008). It is feasible that the observed morphological alterations in the *nhe1* ^−/−^ mucosa are early signs of chronic acid damage, and that the short lifespan of the mice prevented the development of more obvious changes, as observed in the gastric mucosa of carbonic anhydrase IX-deficient mice (Gut et al., 2002; Li et al., 2018). Indeed, a recent publication emphasizes the protective role of NHE1 in the esophagus during environmental stress and suggests an anti-proliferative function in pre-malignancy (Becskeházi et al., 2021).

NHE1 proved to be a major regulator of murine parotid gland fluid secretion *in vivo*, and its loss also reduced parotid gland fluid absorption (Park et al., 2001). The authors also studied the complex compensatory mechanisms that occurred in the *nhe1* ^*−/−*^ parotid acinar cells, demonstrating the difficulties to deduce the role of a transport protein from the phenotype observed in a cell lacking that transport protein (Gonzalez-Begne et al., 2007). In contrast to the studies of parotid and pancreatic acinar cells, in which a strong dependence of pH_i_-maintenance on the expression of NHE1 was demonstrated (Evans et al., 1999; Brown et al., 2003), murine islet cells, although utilizing NHE1 as the major acid extruder during a strong intracellular acid load, did not rely on NHE1 for resting pH_i_-maintenance or for the pH_i_ changes that occur during insulin secretion (Stiernet et al., 2007).

Due to the severe neurological phenotype and growth deficit of the two NHE1-deficient mouse strains (Cox et al., 1997; Bell et al., 1999), we did not attempt to perform intestinal perfusion studies *in vivo*, because perfusion studies obtained in wild type and knockout mice of very different body weight are difficult to interpret. Instead, we made use of the fact that in the rodent intestine, NHE1 was the only basolateral isoform. However, a number of Na^+^-dependent HCO_3_^−^ transporters, which act as base importers, are expressed in the intestine (Damkier et al., 2007). Rabbit duodenal bicarbonate secretion was shown to utilize both a DIDS-sensitive basolateral Na^+^,HCO_3_^−^ cotransporter as well as NHE1 for base supply to the duodenocyte (Jacob et al., 2000). The researchers found that the inhibition of either Na^+^,HCO_3_^−^ cotransport or carbonic anhydrase reduced ouabain-sensitive HCO_3_^−^ flux *in vitro* rabbit duodenal mucosae by approximately 50%, but did not affect 8-Br-cAMP-induced HCO_3_^−^ flux (J (HCO_3_^−^)), suggesting cAMP-mediated upregulation of the alternative pathway. However, inhibition of both Na^+^,HCO_3_^−^ cotransport and either carbonic anhydrase or NHE1 strongly reduced ΔJ (HCO_3_^−^). Therefore, NHE1-mediated proton extrusion is a crucial part of the supply of bicarbonate ions during stimulated HCO_3_^−^ secretion.

### Open Questions and Future Research Opportunities Related to the Role of NHE1 in Gastrointestinal Physiology

As mentioned above, the severe phenotype and early death of the NHE1 null mouse precluded a detailed study of the role of NHE1 in gastrointestinal organ function. To learn more about the involvement of NHE1 in gastrointestinal growth and differentiation, absorption and secretion, in barrier function and microbiome regulation would be of importance, given the fact that NHE1 inhibition is discussed as a strategy for curbing tumor growth and/or its invasive properties in a large variety of organs and tumor cell types (Stock et al., 2012; Harguindey et al., 2013; Meehan et al., 2017; Guan et al., 2018; Hyun et al., 2019; Iorio et al., 2020; Tamtaji et al., 2020; Greco et al., 2021; Mo et al., 2021), as well as for organ protection during ischemia (Jung et al., 2006; Lee et al., 2009; Doods and Wu, 2013; Karmazyn, 2013; Wu et al., 2013; Sasamori et al., 2014), and as an anti-inflammatory and barrier-protective strategy (Khan et al., 2005; Farkas et al., 2010; Wu and Qi, 2012; Yang et al., 2013; Monet et al., 2016; Zhang et al., 2018; Dubaniewicz et al., 2021). However, the recent advancement in the generation and maintenance of epithelium-derived organoids from all gastrointestinal organs with preservation of their organ- and site specific function (Co et al., 2021; Gómez and Boudreau, 2021; Jantaree et al., 2021; Puschhof et al., 2021; Shiota et al., 2021), now opens an avenue to learn more about the role of NHE1 in the development and differentiation pattern of gastrointestinal epithelial cells, about its involvement in transepithelial transport of nutrients and electrolytes, and about its importance in microbial resistance and barrier function. First studies are published that demonstrate a differential expression of acid/base transporters along with absorptive or secretory ion channels or exchangers during the differentiation of human (Yin et al., 2018; Zomer-van Ommen et al., 2018; Zhou et al., 2021) or mouse (Nikolovska et al., 2022) intestinal organoids. Monolayer cultures of gastrointestinal epithelia in predetermined differentiation states with a more homogeneous cell population than present in the full thickness small intestinal crypt-villus or colonic crypt-surface cell epithelium may allow the selective targeting of apical and basolateral NHEs in specialized cellular transport functions, such as anion or mucus secretion, salt absorption, or in barrier maintenance against luminal noxae or pathogens.

## NHE2 in the Gastrointestinal Tract

### Cloning, Regulation and Kinetic Properties of NHE2

Electroneutral Na^+^/H^+^ exchange present in the apical membrane vesicles from rat colon with distinctive kinetic properties suggested the presence of a Na^+^/H^+^ exchanger different than the already identified NHE1 (Rajendran and Binder, 1990). Following this study, a screening of the rat colon cDNA library using NHE1 as a probe identified the presence of Slc9a2 (NHE2) cDNA sequence (Wang et al., 1993). NHE2 was subsequently cloned from rat, rabbit and human intestinal cDNA libraries (Collins et al., 1993; Ghishan et al., 1995; Malakooti et al., 1999; Tse et al., 1993; Wang et al., 1993). The cloned rabbit and rat NHE2 were overexpressed in NHE-deficient cells in two separate studies and its kinetic properties were characterized along with NHE1 and NHE3 (Levine et al., 1993; Yu et al., 1993). The reported values for the Km [Na^+^] and pK [pH] are shown in (Table 2). NHE2 was identified as a Na^+^/H^+^ exchanger with a high amiloride sensitivity and a uniquely high proton affinity both to the intra-and extracellular binding site (Yu et al., 1993; Kapus et al., 1994).

**TABLE 2:** Summary of reported K_m_ [Na^+^] and pK [pH_i_] values for NHE1, NHE2, NHE3, NHE4 and NHE8.

NHE Isoform	Species	K_m_ [Na^+^] mM	pK [pH_i_]	pK [pH_e_]	References
NHE1	Rat	10	6.75	7	Yu et al. (1993)
	Rabbit	15	6.4	ND	Levine et al. (1993)
NHE2	Rat	50	6.9	7.9	Yu et al. (1993)
	Rabbit	18	6.85	ND	Levine et al. (1993)
NHE3	Rat	4.7	6.45	7	Yu et al. (1993)
	Rabbit	17	6.9	ND	Levine et al. (1993)
NHE4	Rat	40	ND	ND	Bookstein et al. (1996)
NHE8	Rat	23	6.5	ND	Xu et al. (2008)

Studies using cloned rat and human NHE2 promoters identified putative binding sites for a number of transcriptional factors (Muller et al., 1998; Malakooti et al., 2001), and found that the NHE2 promoter contains multiple GC-boxes that are targeted by members of the Sp transcription factor family (Muller et al., 1998; Black et al., 2001). A study using transfected renal epithelial cells has identified Sp1 as an activator, and Sp3 and Sp4 as inhibitors of NHE2 transcriptional activation (Bai et al., 2001). In contrast, in intestinal epithelial cells, both Sp1 and Sp3 transcription factors, acted stimulatory on the NHE2 promoter (Hua et al., 2007; Pearse et al., 2007). The epidermal growth factor (EGF) can also regulate NHE2 expression on transcriptional level (Xu et al., 2001; Amin et al., 2011). Promoted NHE2 expression by phorbol 12-myristate 13-acetate (PMA) *via* activation of the PKC and ERK1/2 signaling cascade and the Egr1 transcription factor has been reported as well (Kandasamy et al., 1995; Muthusamy et al., 2012).

Data describing NHE2 protein regulation and kinetics are acquired in studies utilizing exogenous expression of NHE2 in PS120 fibroblasts (a cell line deficient in NHE expression) (Levine et al., 1993; Tse et al., 1994; Cavet et al., 1999; Cavet et al., 2001) or in COS cells (an NHE-deficient CHO cell line) (Yu et al., 1993; Kapus et al., 1994). Besides the uniquely high proton affinity, another interesting finding was the remarkably short half-life of the NHE2 protein (∼3 h) compared to other NHE isoforms (NHE1—24 h, NHE3—14 h) and that it is subject to lysosomal degradation, as determined in PS120 fibroblasts and Caco-2 cells (Cavet et al., 2001). The study has shown that inhibiting either the synthetic pathway or the degradation alters the transport activity of NHE2 (Cavet et al., 2001). Therefore, alterations at the level of gene transcription or translation may be crucial for NHE2 regulation. So far, relatively little is known about the regulation of NHE2 activity. Only few factors have been implied in its regulation, such as serum, fibroblast growth factor (FGF), and protein kinase C (Levine et al., 1993) that act stimulatory, or intracellular ATP depletion that inhibits NHE2 activity by affecting the H^+^ affinity (Levine et al., 1993).

### Pharmacological Inhibition of NHE2

A number of “selective Na^+^/H^+^ exchange isoform 1 inhibitors” have been developed as a possible treatment of cardiac ischemia or as anticancer therapy, and most of them are able to also inhibit NHE2, albeit with different IC_50_ values (Table 1). However, it should be noted that the designated IC_50_ values are derived from studies with exogenous NHE expression in NHE-deficient fibroblasts, and the sensitivity of endogenous NHEs will likely differ (Kiela and Ghishan, 2018). Based on these studies, it has been reported that NHE2 stably transfected in NHE-deficient Chinese hamster ovary (CHO) cells (AP-1) can be inhibited by amiloride and its analogues, with the following inhibition sensitivity: EIPA (IC_50_ = 79 nM) > DMA (IC_50_ = 250 nM) > amiloride (IC_50_ = 1.4 μM) > benzamil (IC_50_ = 320 μM). NHE2 can be inhibited as well by non-amiloride compounds with the following order of sensitivity: clonidine (IC_50_ = 42 μM) > harmaline and cimetidine (both with IC_50_ = 330 μM) (Kiela and Ghishan, 2018; Tse et al., 1993). A number of inhibitors different than amiloride, that offer better sensitivity and allow better isoform separation have been later developed; among them are HOE642 [cariporide; (Counillon et al., 1993)] and HOE694 (Scholz et al., 1993). Both inhibitors have been extensively used for NHE2 studies and allowed partial separation of NHE2 activity from the activity of other isoforms in colonic crypts, where endogenous expression of all NHE isoforms is maintained (Bachmann et al., 2004; Guan et al., 2006). However, due to the overlapping inhibitory concentration for some of the NHE isoforms, and the difficulty to separate the basal from the apical membrane, the assessment of the activity of the different NHE isoforms is still a challenge (Table 1). In two recent publications we studied the activity of the different NHE isoforms in Caco2BBe cell monolayers cultured on transwell filters with relatively high endogenous mRNA expression of NHE1,NHE2, NHE3 and NHE8, but not NHE4 (Yu et al., 2019; Zhou et al., 2021). We were able to successfully separate the activity of the apical NHE isoforms 2,3 and 8 from the basolateral NHE1 using a fluorometric approach with a dual perfusion chamber and the selective addition of Na^+^ and inhibitors to the apical and basolateral side of the cells. In this model, lack of Na^+^ in one perfusate will block the activity of all NHE isoforms in the respective membrane, but will allow the detection of NHE activity in the opposite membrane where Na^+^ perfusion is ongoing. This approach enabled inhibition of the abundant NHE1 activity in the basolateral membrane without interfering with the modest NHE2, NHE3 and NHE8 activities in the apical membrane (Yu et al., 2019; Zhou et al., 2021). In our studies, we have used HOE642, instead of HOE694 since it offers better separation between NHE1, NHE2, and NHE3 with IC_50_ values of 0.05, 3, and 1,000 μM, respectively (Scholz et al., 1995), and we were able to completely inhibit NHE2 activity in the apical membrane using 60 µM HOE642 without interfering with apical NHE3 activity (Yu et al., 2019). Later, however, an inhibition of the NHE8 with 3 µM HOE642 was shown in the same culture model, indicating that the activity attributed to NHE2 in the studies by Yu et al. (2019), and Bachmann et al. (2004) overlaps with the activity of NHE8 which remained masked. Application of the new NHE3 inhibitor, tenapanor (Spencer et al., 2014), which has no inhibitory effect on NHE1, NHE2 or NHE8 (Yu et al., 2019; Zhou et al., 2021), combined with an additional inhibition of NHE8 [3 µM HOE642 (Zhou et al., 2021)] in the apical membrane allowed us to unmask the activity of NHE2 activity in the apical membrane of these cells and correlate it to its relatively high mRNA expression (Zhou et al., 2021). However, it should be noted that the 3 µM HOE642 inhibiting NHE8 activity, is able to inhibit to some degree (∼15%) the activity of NHE2 as well (Paehler Vor der Nolte et al., 2017). Nevertheless, this overlap is even more pronounced with the use of HOE694 and other NHE inhibitors and can probably explain many of the discrepancies related to the relative contribution of different NHE isoforms in different models. In particular, the inhibitory concentration of 10 µM HOE694 that is reported to inhibit NHE8 (Xu et al., 2008) would already inhibit NHE2 to a significant extent (Counillon et al., 1993), and 80 µM of S3226 (an NHE3 inhibitor (Schwark et al., 1998)) would inhibit NHE3, ∼50% of NHE2 (Schwark et al., 1998) as well as rodent NHE1 activity.

Although the application of different inhibitors has offered some insight into the activity of different NHE isoforms, including NHE2, it seems that the overlapping inhibitory profile of most of the applied inhibitors limits their discriminatory potential *in vivo*. The accessibility of gene knockout *in vitro* and *in vivo* models offers additional clarification and their combined usage allows a closer look at the activity and function of certain NHE isoforms in the system of interest.

### Expression of NHE2 in the Gastrointestinal Tract

NHE2 is predominantly expressed in epithelial cells of the gastrointestinal tract (Ghishan et al., 1995; Hoogerwerf et al., 1996; Park et al., 1999; Lee et al., 2000; Rossmann et al., 2001; Narins et al., 2004). However, it is also found outside the GI tract, namely in the kidney (Chambrey et al., 1998; Peti-Peterdi et al., 2000), endometrium and placenta (Johansson et al., 2002; Wang et al., 2003) chondrocytes (Trujillo et al., 1999), inner ear (Bond et al., 1998; Goto et al., 1999), heart, testes, and pituitary gland (Miller et al., 2011). Immunofluorescence analyses have shown apical localization of NHE2 in gastrointestinal epithelial cells (Guan et al., 2006; Rajendran et al., 2015; Aihara et al., 2016). Variations in the NHE2 expression pattern along the crypt-villus axis of the intestine have been observed among different species. For example, in rabbits, NHE2 has been described in the villus brush-border of the small intestine and in the upper half and surface cells of the colonic crypt (Hoogerwerf et al., 1996). Conversely, in the mouse colon, NHE2 is predominantly expressed in the crypt cells (Chu et al., 2002; Bachmann et al., 2004; Guan et al., 2006), which has been functionally confirmed as well (Bachmann et al., 2004; Nikolovska et al., 2022). Due to limited access of a specific antibody, no exact localization of NHE2 in the human gastrointestinal tract has been shown. Using a combination of pH-fluorometry (with dual perfusion to physically separate apical and basolateral membrane) and specific NHE inhibitors, our group has functionally identified NHE2 in the apical membrane of Caco2BBe cells that are often used as a model for intestinal epithelial cells (Yu et al., 2019; Zhou et al., 2021). A model of mouse colonoids designed to resemble different segments of the colonic crypt: 1) colonoids enriched in stem cells, 2) colonoids enriched in transit-amplifying progenitor cells, and 3) differentiated colonoids, has revealed a strong increase of NHE2 mRNA expression in the transit-amplifying progenitor cells (Nikolovska et al., 2022). Figures 1–3 recapitulate the distribution of NHE2 along the gastrointestinal tract, with a focus on gastric cells, small intestinal and colonic epithelial cells.

### NHE2 Activity in the Gastrointestinal Tract

Even though nearly 30 years have passed since the cloning of NHE2 including the first studies characterizing its kinetic properties, the exact physiological role of NHE2 in the gastrointestinal tract remains elusive. Most of the published data were derived from the *nhe2* ^*−/−*^ mice generated by the working group of Gary Shull (Schultheis et al., 1998a). The findings for different parts of the gastrointestinal tract of the *nhe2* ^*−/−*^ mice, revealing the physiological role of NHE2 in the corresponding GI segment, will be discussed here.

Although NHE2 is expressed in the apical membrane of interlobular and main ducts of rodent salivary glands (He et al., 1997), Na^+^ and Cl^−^ absorption and saliva osmolality in *nhe2* ^*−/−*^ mice remained unchanged compared to wild type mice, showing that NHE2 is not involved in Na^+^ reabsorption by the salivary gland duct epithelium, and its loss is compensated by an increased expression of ENaC (Park et al., 1999). Furthermore, acid-loaded acinar cells from *nhe2* ^*−/−*^ and wild type mice had a comparable pH recovery rate, excluding a role of NHE2 in pH_i_ regulation of these cells (Evans et al., 1999) as well.

In exocrine pancreas, pancreatic acinar cells secrete a NaCl-rich fluid, which is later modified to a bicarbonate-rich pancreatic juice by the ductal epithelial cells. A participation of active apical Na^+^/H^+^ exchangers in this process was assumed, specifically the contribution of NHE2, which was found to be expressed in both acinar and pancreatic duct cells (Zhao et al., 1994; Marteau et al., 1995; Lee et al., 2000; Brown et al., 2003). However, pancreatic acinar cells isolated from *nhe2* ^*−/−*^ and wild type mice displayed no difference in the kinetics of pH_i_ recovery (Brown et al., 2003), nor did the luminal Na^+^-dependent H^+^ efflux in *nhe2* ^*−/−*^ ducts differ from the one detected in wild type (Lee et al., 2000), suggesting that NHE2 plays no role in exocrine pancreatic juice secretion.

In the stomach, NHE2 is expressed in all three types of gastric epithelial cell (Seidler et al., 1989) (Figure 1). Upon generation of *nhe2* ^*−/−*^ mice, a gastric phenotype was reported, with reduced numbers of parietal and chief cells (Schultheis et al., 1998a) and occurrence of diffuse corporal gastritis progressing to atrophic gastritis with chronic achlorhydria (Boivin et al., 2000). The number of parietal cells in these mice was reduced and they were found at different stages of necrosis (Schultheis et al., 1998a). However, the matured *nhe2* ^*−/−*^ parietal cells were functionally able to secrete acid, suggesting that NHE2 is not directly involved in the process of acid secretion, but necessary to keep the viability of the parietal cells, and probably involved in their differentiation, which has not been directly addressed. NHE2 was predicted to be activated during stimulation of acid secretion in response to the increased alkalinity on the basal side of the gastric epithelium (Yu et al., 1993). However, this would mean that NHE2 is basolaterally expressed in gastric epithelial cells, which has been suggested but not proved immunohistochemically (Rossmann et al., 2001). On the contrary, an apical expression of NHE2 in gastric surface and pit epithelial cells has been demonstrated (Xue et al., 2011; Aihara et al., 2016). The apically located NHE2 encounters a highly acidic lumen, which makes it unsuitable to participate in pH_i_-regulation of the gastric surface cells. Later findings suggested a novel role of NHE2 in gastric epithelial restitution (Xue et al., 2011; Aihara et al., 2016; Paehler Vor der Nolte et al., 2017). Xue at al. demonstrated that active NHE2, but not NHE1, is required for mouse gastric epithelial restitution, and that it is necessary for TFF (trefoil factor)-mediated epithelial repair (Xue et al., 2011). Activation of NHE2 could be triggered by a rise in surface pH that occurs after damage. The authors suggest that in the scenario of very acidic extracellular pH (pH4) and physiological intracellular pH (pH7) the apical NHEs are either inactive or operate in reverse mode. In this case, active apical NHE2 would import extracellular H^+^ ions contributing to alkalization of the surface pH and promoting epithelial repair. However, a surface pH increase upon damage was observed in the gastric epithelium even in absence or inhibition of NHE2 (Xue et al., 2011), meaning that the change of surface pH is not the mechanism by which NHE2 stimulates gastric repair. The possibility that NHE2 shifts to another location during cell migration in a similar manner to NHE1 (Stock and Schwab, 2006; Clement et al., 2012), potentially triggering pro-migratory signaling pathways remains open. The effect of the intracellular pH, which would be affected in absence of NHE2, was not addressed by this study. A subsequent investigation from the same group showed that even 30 days upon injury, NHE2 remains downregulated in the regenerated gastric epithelium (Aihara et al., 2016), which raises the question of whether the downregulation of NHE2 expression could be an indication of abnormal ion transport activity in the healed epithelium. Indeed, *nhe2* ^*−/−*^ mice had a lower basal short circuit current (I_sc_), and a decreased I_sc_ recovery in response to 0.5 M NaCl-induced gastric epithelial damage was observed after NHE2 inhibition (Matthis et al., 2020). The study addressed the pH_i_ in gastric organoids during restitution, and found that the pH_i_ was reduced in migrating cells, but it was later recovered during restitution (Matthis et al., 2020). Thus, NHE2 may be important for restitution by helping regulate pH_i_. Our group has used the RGM1 cell line (rat gastric mucosal cells) (Kobayashi et al., 1996) to investigate the role of NHE2 in gastric epithelial restitution. Due to the low endogenous NHE2 expression in these cells, the cells were transduced with NHE2 (transduction efficiency was 70–80%), which resulted in a robust increase in the steady-state pH_i_, as well as high NHE2-mediated pH_i_-recovery rates in the transduced RGM1 cells, but their migration rate in a wound healing assay did not differ from the control cells under basal (pH7.4) conditions. However, after acid preincubation, a significant decline in the migratory speed of NHE2 expressing cells was observed, a result that could be abolished by inhibition of NHE2 with 50 µM HOE642 (Paehler Vor der Nolte et al., 2017). Beside the demonstrated role of NHE2 in gastric restitution, the molecular mechanism of how NHE2 regulates the process remains unidentified.

NHE2 activity was detected in the brush border of rabbit ileum and human duodenum (Wormmeester et al., 1998; Repishti et al., 2001). In mouse duodenum, NHE2 is an active participant in the maintenance of intracellular pH_i_ of the enterocytes (Praetorius et al., 2000). However, despite the high mRNA abundance of NHE2 in the intestine, the *nhe2* ^*−/−*^ mice show no obvious intestinal phenotype unlike the severe diarrhea observed in *nhe3* ^*−/−*^ mice (Schultheis et al., 1998a). The net Na^+^ and Cl^−^ absorption rate (Gawenis et al., 2002), and the fluid absorptive rate (Xia et al., 2014) in jejuna of wild type and *nhe2* ^*−/−*^ mice was similar, which shows that NHE2 does not act as Na^+^ absorptive mechanism in the intestine, as it has been shown for other epithelial cells in the salivary glands and the pancreas (Ledoussal et al., 2001a; Luo et al., 2001; Park et al., 2001). NHE2 does not compensate for the loss of NHE3 in the *nhe3* ^*−/−*^ intestine, since NHE2 mRNA expression remained unchanged in *nhe3* ^*−/−*^ mice, and the residual, EIPA-sensitive Na^+^ absorption that remained is unlikely to be mediated by NHE2 since it was reduced by elevated cAMP (Gawenis et al., 2002) (which in *vitro* studies increased NHE2 activity (Kandasamy et al., 1995)). Also, double *nhe2* ^*−/−*^ *nhe3* ^*−/−*^ knockout mice were not affected by the additional loss of NHE2, and showed no additional worsening of the systemic acid-base balance, or diarrhea observed previously in the *nhe3* ^*−/−*^ mice (Ledoussal et al., 2001b). Despite the negligible role of NHE2 in Na^+^ absorption in the small intestine, its high expression abundance should not be overseen and its function in pH regulation or cell homeostasis remains to be determined, as well as the mechanism that compensates for its loss in the small intestinal segments.

The role of NHE2 in epithelial restitution in the small intestine was analyzed using mesenteric ischemia *in vivo* to injure the ileum of *nhe2* ^*−/−*^ and wild type mice (Moeser et al., 2008). The study showed increased small intestinal permeability during the post-ischemic recovery in *nhe2* ^*−/−*^ ileum compared to wild type. The failed reestablishment of the tight junctions post injury, especially disrupted occludin and claudin-1 localization patterns in the *nhe2* ^*−/−*^ tissue, was offered as a mechanistic explanation (Moeser et al., 2008). Our group has used Caco2BBe cells to address the impact of NHE2 on epithelial restitution, and we found that Caco2BBe cells upon “wounding” of the monolayer exhibit “sheet” migration, which is significantly increased in Caco2BBe cells with downregulated NHE2 expression (Nikolovska et al., 2018). In our model we have shown that the lack of NHE2 in Caco2BBe cells leads to a significant decrease of the intracellular pH_i_ (Yu et al., 2019; Zhou et al., 2021; Nikolovska et al., 2022), which might influence the generation of the tight junction network or different signaling pathways and therefore affect cell migration. This, however, remains to be further investigated.

One study has shown that the luminal pH in different segments of the *nhe2* ^*−/−*^ mice is acidic, which is likely a secondary phenomenon, because the absence of an active Na/H exchanger in the luminal membrane should result in less proton extrusion (Engevik et al., 2013). In the colon of *nhe2* ^*−/−*^ mice *in vivo*, we found a juxtamucosal surface pH that was similar to that of wild type littermates (Nikolovska et al., 2022). The fluid absorption rate in mid-distal colon of *nhe2* ^*−/−*^ and wild type mice was similar. Only after application of the NHE3 specific inhibitor tenapanor (Yu et al., 2019; Tan et al., 2021), we were able to show a minor involvement of NHE2 in colonic fluid absorption (Nikolovska et al., 2022). Neither immunofluorescence analysis nor mRNA expression data showed an increase of NHE3 in the mid-distal colon of *nhe2* ^*−/−*^ mice, albeit the NHE3 immunoreactivity area was extended more deeply into the cryptal mouth region in *nhe2* ^*−/−*^ colon in contrast to wild type mid-distal colon (Nikolovska et al., 2022). In a cell model of Caco2BBe cells, we identified a compensatory effect of NHE8 in cells lacking NHE2 (Zhou et al., 2021), but this was not functionally studied in the *nhe2* ^*−/−*^ intestine. Both Guan et al. (Guan et al., 2006) and we found a significant role of NHE2 in pH_i_ maintenance in the base of the colonic crypts (Bachmann et al., 2004; Nikolovska et al., 2022). pH_i_ fluorometry of isolated mid-distal colonic crypts showed a pH_i_ gradient along the cryptal axis with more acidic values at the base of the crypt sequentially shifting to more alkaline pH_i_ values towards the cryptal surface. In the *nhe2* ^*−/−*^ crypts isolated from the same region, a significantly lower pH_i_ was detected in the middle segments of the crypt compared to the wild type (Nikolovska et al., 2022). This suggests that NHE2 is an essential pH_i_ regulator in the transit amplifying progenitor zone of the colonic crypt, and that an increased NHE2 activity, associated with an increase in pH_i_, is an early event during progenitor cell proliferation and differentiation. Indeed the differentiation program of NHE2-silenced colonic epithelial cells was altered with shift from the absorptive towards the secretory lineage (Nikolovska et al., 2022). The observed thicker mucus layer, longer crypts and an expanded brush border membrane zone of NHE3 abundance in the *nhe2* ^*−/−*^ colon are further indications of an impact of NHE2 activity on the orderly differentiation in the colonic mucosa (Nikolovska et al., 2022). An open question is how the lower pH_i_ in the colonic progenitor cells affects their differentiation program. We have observed differences in the Wnt/Notch signaling pathway in colonocytes lacking NHE2 that are in line with the altered differentiation program (Nikolovska et al., 2022). However, the question of whether there is a direct interaction with the signaling molecules, or the signaling pathway is altered, due to the change in pH_i_ as previously shown for other models (Ulmschneider et al., 2016) remains unanswered. Previous publications have shown that NHE2 is transcriptionally regulated by EGF, and that EGF stimulation leads to increased NHE2 activity *in vivo* and *in vitro*, both in a species and age related manner (Xu et al., 2001). In subconfluent Caco2BBe cells lacking NHE2, EGF did not affect cell proliferation nor did it initiate the ERK1/2 signaling cascade (Zhou et al., 2021). The interaction of NHE2 with the different signaling cascades is probably conducted *via* its C terminus, where the proline rich regions interact with the SH3 domain of signaling protein kinases, and coupling of proteins *via* their SH3 domains has been implicated in a variety of functions, including regulation of cell proliferation (Broome and Hunter, 1996; Erpel et al., 1996). The SH3-binding domain plays an important role in the EGFR signaling (Liu et al., 1997) and Wnt signaling pathway (Yokoyama and Malbon, 2009), therefore the absence of NHE2 C-terminus coupling to the SH3 domains could be the reason for decreased activation of these signaling pathways, although many other alternative proteins can overtake this functions. It is more likely that the pH_i_ regulated by NHE2 in the apical membrane is the crucial factor by which NHE2 influences signaling pathways, as was shown for CFTR in the intestinal stem cells (Strubberg et al., 2018) or the Na^+^/H^+^ exchanger DNhe2 in *Drosophila* adult follicle stem cells (Ulmschneider et al., 2016).

Addressing the role of NHE2 in the gastrointestinal epithelial homeostasis has been hampered by the absence of models that accurately assess its expression and activity in different epithelial compartments, as well as the absence of a specific antibody. The ability to generate intestinal organoids representing distinct segments of the GI tract at different differentiation stages will allow further clarification of the physiological role of NHE2. However, the requirement of constant growth factor supplementation for organoid culture maintenance represents an obstacle to using organoid cultures in signaling pathway studies. NHE2 in the pathophysiology of the gastrointestinal tract.

### NHE2 in the Pathophysiology of the Gastrointestinal Tract

The involvement of NHE2 among other NHE isoforms in the pathophysiology of the gastrointestinal tract has been addressed scarcely in several reviews (Ghishan and Kiela, 2014; Gurney et al., 2017; Das et al., 2018; Xu et al., 2018; Cao et al., 2019). Due to the lack of diarrhea and an intestinal phenotype in the *nhe2* ^*−/−*^ mice (Ledoussal et al., 2001b), but observed alterations in the gastric epithelium (Schultheis et al., 1998a), NHE2 was mentioned in the pathophysiology of the stomach, where it was found necessary for the healing of the gastric mucosa post ulceration (Aihara et al., 2016). Enteric infections triggered by bacterial or viral infection causing diarrhea are often brought into context of NHE activity due to the impaired NaCl and fluid absorption (Gurney et al., 2017). *In vitro* treatment of a number of intestinal epithelial cell lines (Caco2BBe, HT29 cells, and T84 cells) with the enteropathogenic *E. coli* (EPEC) resulted in increased expression and activity of NHE1 and NHE2, but a decreased activity of NHE3 (Hecht et al., 2004). In contrary, in rotavirus infected patients the expression of NHE2 was decreased along with NHE3 (Baetz et al., 2016).

Another pathophysiological condition where NHE activity (in particular NHE3) is extensively investigated is inflammatory bowel disease (IBD). NHE2 expression and activity was affected by inflammatory markers, as *in vitro* data using Caco2 cells treated with inflammatory cytokines, TNF-α (tumor necrosis factor α) and IFN-γ (interferon γ) showed reduced NHE2 expression and activity (Rocha et al., 2001; Amin et al., 2011). Although this would imply that the increased gradient of both TNFα and IFNγ in IBD can lead to a decrease in NHE2 expression, in colon biopsies from IBD patients NHE2 expression and activity were not altered (Sullivan et al., 2009; Farkas et al., 2010). However, in rats with TNBS-induced colitis, both mRNA and protein NHE2 expression were reduced (Soleiman et al., 2017). NHE2 was activated and played a major role in butyrate-dependent Na^+^ absorption (Rajendran et al., 2015) in the inflamed colon of DSS treated rats, but in absence of inflammation, this role was conducted by NHE3.

The involvement of NHE2 in epithelial restitution in the gastric epithelium and in the postischemic intestine indicates its role in migration of gastrointestinal epithelial cells, but this has not been investigated so far.

## NHE3 in the Gastrointestinal Tract

### Expression and Localization in Gastric and Intestinal Epithelia

In 1985, Ganapathy and Leibach (1985) hypothesized that intestinal peptide absorption depends on a proton gradient across the brush border membrane and that a Na^+^/H^+^ exchange mechanism in the apical membrane in conjunction with the Na^+^/K^+^-ATPase in the basolateral membrane would generate and maintain this proton gradient. In 1992, NHE3, encoded by the *Slc9a3* gene, was cloned and sequenced from rat (Orlowski et al., 1992) and rabbit (Tse et al., 1992) where its expression in the kidneys, the small intestine (mainly jejunum and ileum) and the ascending colon pointed to its functional role in Na^+^ absorption by intestinal and renal epithelial cells. About another decade later, it turned out that intracellular pH (pH_i_) functionally couples the absorption of Na^+^ and dipeptides, which confirmed the original hypothesis by Ganapathy and Leibach (1985): in the intact nematode *Caenorhabditis elegans*, the ortholog of mammalian NHE3, CeNHX2, is expressed exclusively in the apical membrane of intestinal epithelial cells. By removing protons from the cytosol, CeNHX2 stabilizes pH_i_ including the proton gradient required to drive the activity of the H^+^-oligopeptide symporter OPT2 (Nehrke, 2003). Secondly, in human intestinal epithelial cells (Caco-2), the coordinated activity of NHE3 and the intestinal di/tripeptide transporter hPepT1 allows for a smooth peptide and peptide-like drug transport across the luminal membrane (Anderson et al., 2003). While the presence and physiological role of NHE3 in the luminal membranes of enterocytes of both the small intestine and the colon are undeniable, its expression and function in gastric epithelia is anything but clear (Figures 1–3). NHE3 is not expressed in the stomach of rabbits (Rossmann et al., 2001), has been shown to localize in basolateral membranes of human and guinea pig gastric surface mucous cells (Kulaksiz et al., 2001), and is present in the apical membrane of rat gastric parietal cells where it colocalizes with the β-subunit of the gastric H^+^-K^+^-ATPase (Orlowski et al., 1992; Kirchhoff et al., 2003). Because of these inconsistencies across the species regarding gastric NHE3 expression and location, it is hardly possible to come up with a commonly accepted idea of NHE3’s physiological role in the stomach. For that reason, we focus on intestinal NHE3 in the following paragraphs.

### Physiological Function(s) of Intestinal NHE3 and its Involvement in Diarrheal Diseases

Independently of the above-mentioned coupling with di-/tripeptide absorption, NHE3 activity represents the major mechanism of intestinal Na^+^ absorption accompanied by mostly paracellular water uptake (Karasov, 2017). It thus contributes significantly to body fluid and blood pressure homeostasis as well as acid-base regulation (Pedersen and Counillon, 2019; Kovesdy et al., 2021; Zhuo et al., 2021) which is supported by a number of studies using elaborate NHE3 knockout mouse models, such as tissue-specific (Dominguez Rieg et al., 2016) or tamoxifen-inducible intestinal epithelial cell-specific NHE3 knockout mice (Xue et al., 2020), or specific drugs such as the first-in-class NHE3 inhibitor tenapanor (Spencer et al., 2014; Yu et al., 2019; Zhou et al., 2021). Treating NHE2 knockout mice with tenapanor revealed that NHE2 contributes to colonic fluid absorption only marginally, if at all (Nikolovska et al., 2022). This recently published observation does not only support the finding that the diarrheal phenotype of NHE3 knockout mice is not further aggravated in NHE3/NHE2 double knockout mice (Ledoussal et al., 2001b), but clearly represents one more piece of evidence that confirms NHE3 as the major player in Na^+^ and fluid absorption. The necessity of a properly functioning NHE3 becomes also manifest in several pathologies resulting from its malfunction (Cao et al., 2019). Accordingly, NHE3 knockout mice suffer from low blood pressure and metabolic acidosis, to be ascribed primarily to the NHE3 deficient kidney though, as well as intestinal malabsorption and diarrhea (Schultheis et al., 1998b). The classical, i.e. non-syndromic, congenital sodium diarrhea is caused by either an autosomal recessive loss-of function-mutation in the *Slc9a3* gene or by a dominant gain-of-function mutation in GUCY2C, the gene encoding intestinal receptor guanylate cyclase C (GCC). The latter is called “secondary NHE3 deficiency”. The resulting increase in cGMP downregulates NHE3 activity *via* protein type II cGMP dependent kinase (PKG II) and the cAMP-dependent protein kinase PKA (Müller et al., 2000; Janecke et al., 2016). Excessive stimulation of genetically unaltered GCC by bacterial heat-stable enterotoxin STa has the same effect. STa is secreted by enterotoxigenic *Escherichia coli* (ETEC) and represents the major cause for acute secretory diarrhea, including traveller’s diarrhea, in developing countries with insufficient sanitation and inadequate supply of clean water (Weiglmeier et al., 2010). cGMP does not only stimulate PKGII and PKA but it also inhibits phosphodiesterase 3 (PDE3). When inhibited, PDE3 cannot hydrolyze cAMP. cAMP then accumulates and stimulates PKA in addition to cGMP, resulting in a strong inhibition of NHE3 (He and Yun, 2010). Although enteropathogenic *E. coli* (EPEC) do not release proteins defined as classic toxins they still induce a decrease in NHE3 activity and cause life-threatening diarrhea in newborns and young children (<3 years). To date, the underlying mechanism has not been fully elucidated yet. However, specifically in humans the interaction of NHE3 with the PKA-dependent E3 ubiquitin ligase Nedd4-2 potentiates NHE3 inhibition and exacerbates the severity of diarrhea (Hecht et al., 2004; Jenkin et al., 2022).

Independently of GCC activity and PDE3 inhibition, the cholera toxin, an exotoxin produced by *Vibrio cholerae*, leads to high cAMP levels. Once in the cytosol, the CTA1 subunit of the toxin catalyzes the ADP-ribosylation of the trimeric Gsα component of the adenylate cyclase AC. AC remains in its GTP-bound state and is permanently active to produce cAMP, which inhibits NHE3 activity and expression (Subramanya et al., 2007; Bharati and Ganguly, 2011).

*Clostridium difficile* toxin B also decreases NHE3 activity, mainly due to a Rho-GTPase-sensitive redistribution of NHE3 away from the plasma membrane (Hayashi et al., 2004; Engevik et al., 2015).

The physiological impact of NHE3, like that of virtually all membrane transporters, should not be considered in isolation because its function cannot be understood without comprehensive knowledge of the other major and relevant, functionally interwoven transporters and ion channels located in the plasma membrane. Thus, it is the functional coupling of NHE3 and the apically located Cl^−^ conducting CFTR (cystic fibrosis transmembrane conductance regulator) including their shared - not necessarily in the same direction - regulation by cGMP, PKA and cGMP dependent kinases (cGK) that account for the severity of the mentioned, Na^+^ driven osmotic diarrhea (Arshad and Visweswariah, 2012; Gurney et al., 2017; Liu et al., 2020). While NHE3-mediated Na^+^ absorption is decreased, CFTR-mediated Cl^−^ secretion is increased. This leads to an accumulation of both of these osmotically active ions in the intestinal lumen resulting in osmotic diarrhea. Apart from that, NHE3 cooperates with the Cl^−^/HCO_3_^−^ exchanger SLC26A3, also known as DRA (downregulated in adenoma), to mediate the absorption of Na^+^ and Cl^−^ (Höglund et al., 1996; Melvin et al., 1999; Jacob et al., 2002). Especially along the rodent colonic axis, NHE3 and DRA are expressed differently in different segments. In the proximal colon, NHE3 activity maintains an acidic microenvironment at the mucosal surface, which could potentially drive both the absorption of short-chain fatty acids (SCFA^−^) mediated by a yet-to-be-identified apical SCFA^−^/HCO_3_^−^ exchange mechanism and the nonionic diffusion of protonated SCFA. In the midcolon, NHE3 and DRA coexist in the apical membranes of the same cells and cooperate: they import Na^+^ and Cl^−^ from the lumen across the apical membrane and extrude H^+^ and HCO_3_^−^ (= CO_2_ and H_2_O) (Talbot and Lytle, 2010).

Inflammatory bowel diseases, commonly classified into ulcerative colitis (UC) and Crohn’s disease (CD), are chronic inflammatory disorders. Although the etiology of these multifactorial disorders is complex as it includes a combination of genetic, immunological, environmental and gut microbial factors (Shouval and Rufo, 2017; Zhang et al., 2017), both a significant contribution of electrolyte malabsorption to IBD associated diarrhea (Binder, 2009; Priyamvada et al., 2015; Magalhães et al., 2016) and a considerable share of NHE3 therein is beyond all question (Siddique et al., 2009; Sullivan et al., 2009; Yeruva et al., 2010; Lenzen et al., 2012; Anbazhagan et al., 2018). NHE3 activity is decreased in IBD patients, either because of decreased NHE3 protein levels as found in both UC and CD patient biopsies (Sullivan et al., 2009) with reduced NHE3 mRNA seen only in CD biopsies (Siddique et al., 2009), or independently of its expression, i.e., without any changes in mRNA, protein and surface expression levels as found in biopsies of UC patients (Farkas et al., 2010; Yeruva et al., 2010; Yeruva et al., 2015). Although evidence regarding the underlying mechanism(s) has remained rather inconsistent up to this point, the data obtained from IBD patients and murine models leave no doubt: impaired NHE3 function fundamentally contributes to the pathogenesis of diarrhea in IBD. Beyond that, the fact that the findings are so heterogeneous but still - each one individually - trustworthy point to a complex regulation of both the expression and activity of NHE3.

### Regulation and Trafficking of NHE3

Long-term (chronic) and short-term (acute) regulation of NHE3 activity can be distinguished (He and Yun, 2010), with gene transcription representing the major mechanism of long-term regulation (Malakooti et al., 2011; Muthusamy et al., 2018), while acute regulation within minutes up to hours includes 1) (de)phosphorylation by various kinases (Chen et al., 2015) and phosphatases ((Dynia et al., 2010), 2) dynamic interaction with numerous proteins (Hayashi et al., 2002), and 3) trafficking between the plasma membrane and different intracellular compartments (Alexander and Grinstein, 2009).

### Regulation at Transcriptional Level

**FIGURE 4:**
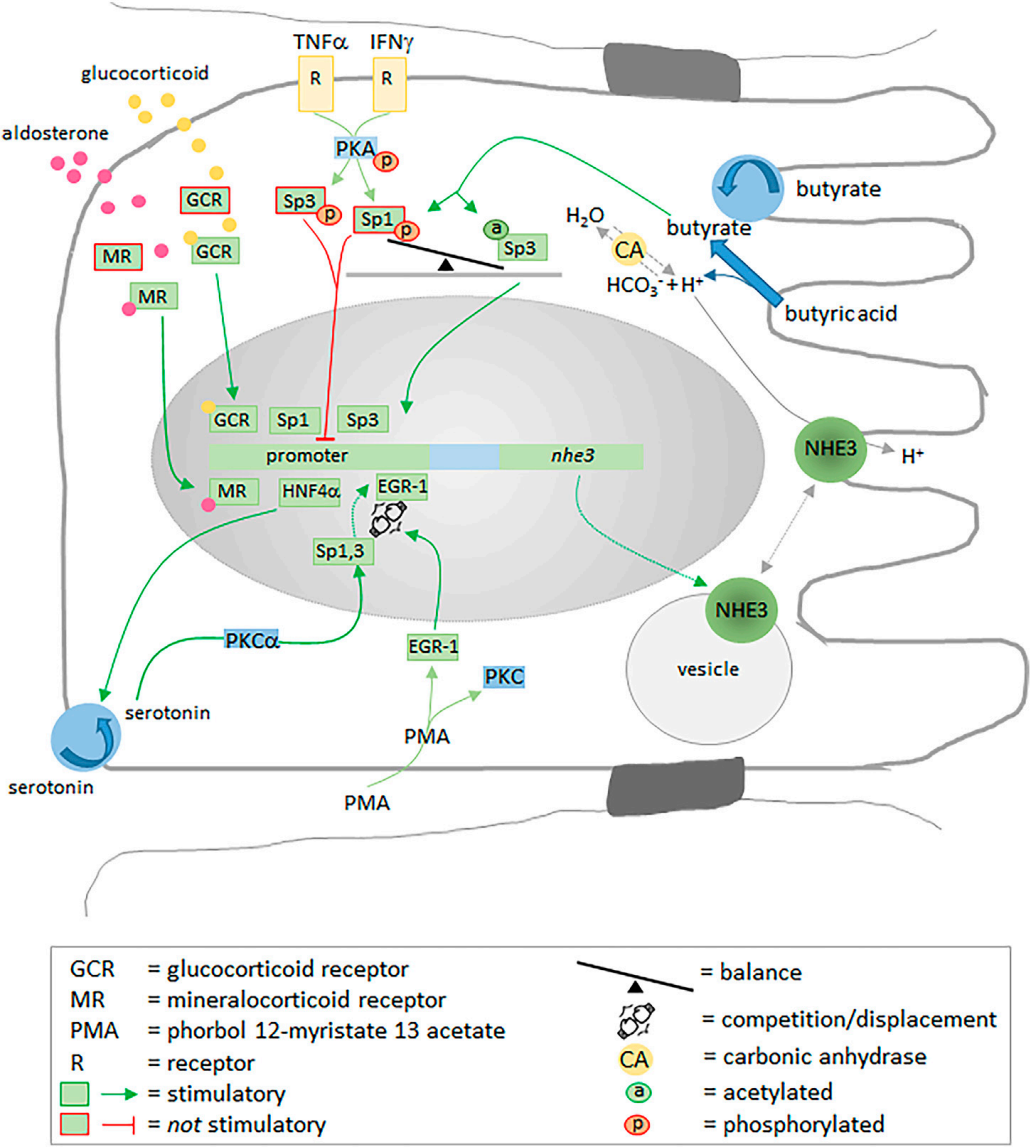
Transcriptional regulation of NHE3 expression. Numerous transcription factors including Sp1, Sp3, EGR-1 and HNF4a, as well as the stimulated glucocorticoid and mineralocorticoid receptors stimulate the expression of NHE3 by binding to promoter motifs. Phosphorylation of Sp1 and Sp3 inhibits their interaction with the promoter. For more detailed information, please see main text.

Glucocorticoids (Kandasamy and Orlowski, 1996; Yun et al., 1993), aldosterone (Cho et al., 1998), metabolic acidosis (Lucioni et al., 2002), and butyrate (Kiela et al., 2007; Musch et al., 2001) activate or enhance the transcription of NHE3 (Malakooti et al., 2011), and the transcription factors Sp1 and Sp3 play central roles in regulating the transcription process (Amin et al., 2007) (Figure 4). Being potentially dependent on Ser/Thr kinases, dephosphorylation of Sp1/Sp3 enhances their binding affinity to the promoter (Kiela et al., 2001). Interestingly, although leading to an increased phosphorylation of Sp1, butyrate stimulates NHE3 gene transcription by acetylating Sp3 (Figure 4). The fact that Sp3 is a more potent inducer of NHE3 gene transcription than Sp1 points to the balance between Sp1 and Sp3 as the critical, regulatory variable (Kiela et al., 2007). Another transcription factor, the early growth response gene product 1 (EGR-1), also stimulates NHE3 mRNA expression. It can be stimulated by phorbol 12-myristate 13 acetate (PMA), a diacylglycerol analogue that stimulates protein kinase C (PKC)-dependent signaling pathways. The PMA-induced, EGR-1-mediated upregulation of endogenous NHE3 mRNA expression, however, does not depend on PKC and relies on the displacement reaction at one of the NHE3 promoter’s binding motifs: binding of EGR-1 displaces Sp1 and Sp3 (Malakooti et al., 2006). Secretion of pro-inflammatory cytokines, such as IFN-γ and TNF-α, contributes considerably to diarrhea associated with IBD, also by the inhibition of NHE3 (Rocha et al., 2001). Both IFN-γ and TNF-α suppress NHE3 promotor activity *via* PKA-mediated phosphorylation of Sp1 and Sp3 (Amin et al., 2006). Moreover, the DNA binding affinities of Sp1 and Sp3 to the Sp1/Egr-1 motif of the NHE3 promoter can be modulated by the neurotransmitter serotonin, probably *via* a PKC-α signaling pathway (Amin et al., 2009). Serotonin is strongly involved in the regulation of gastrointestinal motility (Kendig and Grider, 2015; Del Colle et al., 2020), secretion and absorption (Crowell, 2004), and decreases in the mucosal serotonin level, serotonin transporter mRNA and serotonin transporter immunoreactivity have been associated with IBD (Coates et al., 2004) and carcinoid tumors (Zuetenhorst et al., 2004). Recently, the hepatocyte nuclear factor-4α (HNF4α), a transcription factor regulating the expression of a number of hepatic genes, has been shown to directly regulate NHE3 promotor activity and maintain its expression in the intestine (Muthusamy et al., 2018). At this point, it is worth mentioning that mutations of HNF4α or a long-term reduction of its activity are supposed to result in IBD, possibly also by modifying claudin-15 expression and weakening the mucosal integrity (Darsigny et al., 2009; Babeu and Boudreau, 2014). Particularly interesting is a direct correlation between HNF4α and the serotonin transporter in intestinal epithelial cells: intestine-specific HNF4α knockout in mice causes a drastic reduction in the serotonin transporter and can be related to IBD (Holton et al., 2020). Hence, HNF4α promotes transcription of NHE3 directly by regulating its promoter activity and indirectly *via* supporting serotonin uptake and thus the DNA binding affinities of Sp1 and Sp3.

### Regulation at (post)translational Level and by Trafficking

**FIGURE 5:**
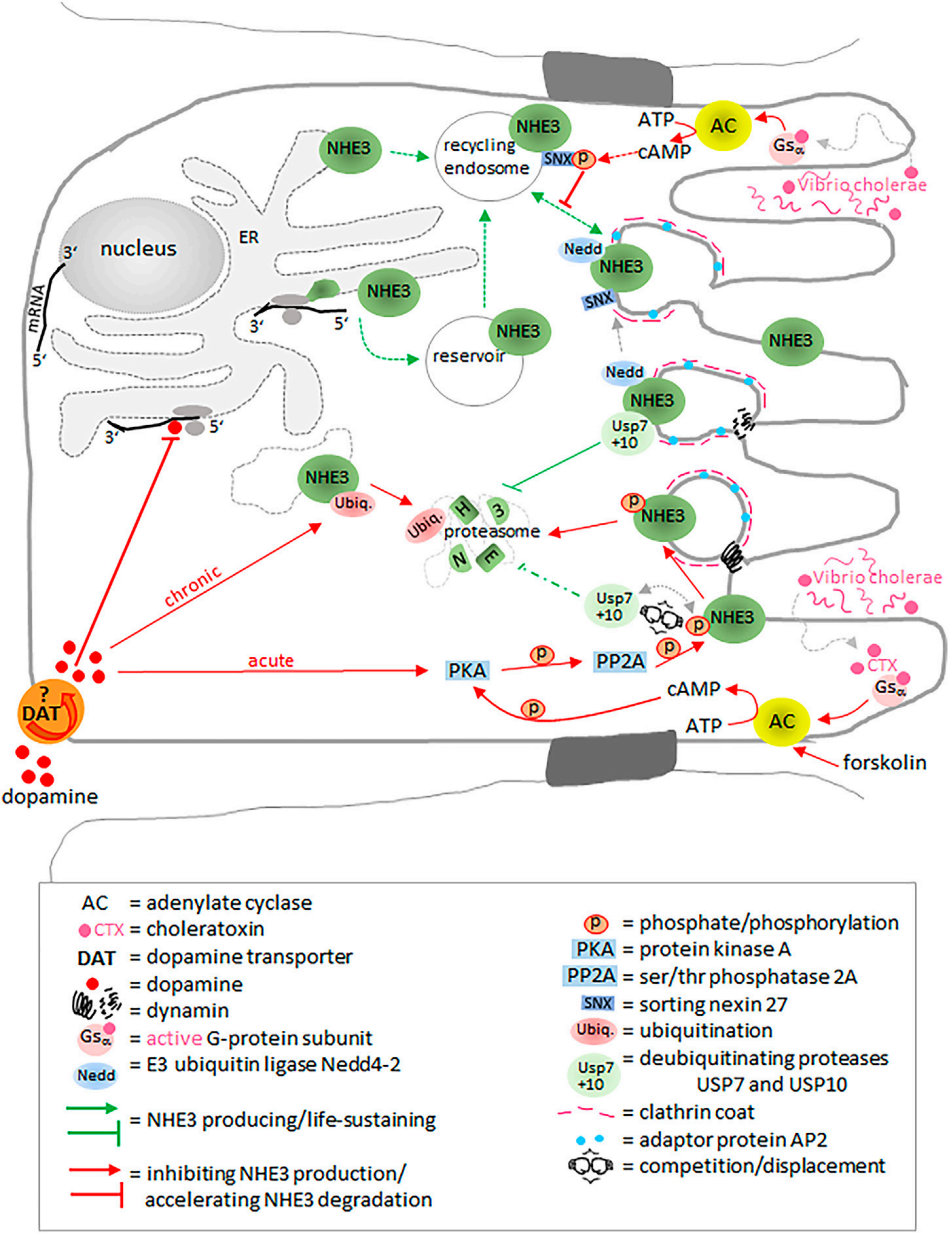
NHE3 regulation at (post)translational level and by trafficking. Dopamine inhibits the translation process and accelerates proteasomal NHE3 degradation by ubiquitination or phosphorylation. The ubiquitin-specific proteases USP7 and USP10 additively prevent protea-/endosomal degradation. Cholera toxin leads to phosphorylation of sorting nexin 27 and NHE3. It thus inhibits trafficking to the membrane and accelerates degradation, respectively. E3 ubiquitin ligase Nedd4-2 mediates removal of NHE3 from the plasma membrane by endocytic internalization without NHE3 being degraded. Please see main text for more detailed information.

To date, not much is known about the translational regulation of NHE3 in the intestinal epithelia. However, in the rat kidney and in OKP cells, dopamine has been shown to decrease NHE3 translation by acting on *cis*-sequences in the 5′-untranslated region of the NHE3 mRNA (Figure 5) (Hu et al., 2013). In addition, chronic application of dopamine increases NHE3 degradation by ubiquitination followed by proteasomal degradation (Hu et al., 2013), while acute dopamine effects include a reduction in the NHE3/phosphorylated NHE3 ratio mediated by protein kinase A (Hu et al., 2001) and serine/threonine phosphatase 2A (Bobulescu et al., 2010), resulting in endocytosis of NHE3 *via* clathrin-coated vesicles (Chow et al., 1999) in a dynamin- and adaptor protein AP2-dependent manner (Collazo et al., 2000). Trafficking between plasma membrane and compartments is a common method of acute regulation. As for NHE3, trafficking represents a temporary compartmentalization of transport activity, i.e., NHE3 remains active in recycling endosomes (D'Souza et al., 1998; Kovesdy et al., 2021). In addition to a constitutively recycling NHE3 population, another intracellular population seems to be located in storage compartments, potentially waiting to be recruited to the cell surface when acutely needed, e.g., in order to reabsorb Na^+^ in proximal renal tubules (Alexander et al., 2005; Alexander and Grinstein, 2009). In humans and non-human primates, removal of NHE3 from the plasma membrane by endocytic internalization can be mediated by the E3 ubiquitin ligase Nedd4-2, without NHE3 being degraded (No et al., 2014). Two deubiquitinating enzymes (DUBs), the ubiquitin-specific proteases USP7 and USP10, additively prevent protea-/endosomal degradation and thus retain NHE3 half-life stability (Han and Yun, 2020). Silencing of USP7 and USP10 causes strong increases in both the co-localization and co-immunoprecipitation of NHE3 with the small GTPases Rab5a and Rab7 ((Han and Yun, 2020). Rab5a drives the maturation from the early to the late endosome (Huotari and Helenius, 2011). Stimulation of protein kinase A by forskolin *via* cAMP leads to an increased NHE3 phosphorylation and decreases the binding of USP7 and USP10 to NHE3 resulting in its enhanced ubiquitination and inhibition (Han and Yun, 2020).

### Regulation by Molecular Interaction With Adapter Proteins

Cholera toxin (CT) secreted by *Vibrio cholerae* provokes diarrhea not only by increasing Cl^−^ secretion through CFTR and decreasing Na^+^ absorption through NHE3, both in a cAMP/PKA-dependent manner, but also by inhibiting trafficking of NHE3 from early endosomes to the plasma membrane (Singh et al., 2018). Sorting nexin 27 (SNX27) is an early endosomal protein containing a PDZ binding motif. It binds and regulates exocytosis of NHE3 from the early endosome to the plasma membrane (Singh et al., 2015). CT phosphorylates an amino acid in the PDZ domain of SNX27 and thus inhibits SNX27-mediated trafficking of NHE3 to the plasma membrane (Singh et al., 2018).

The acute inhibition of NHE3 by cAMP/protein kinase A type II (PKAII) or cGMP/cGMP-dependent protein kinase type II (cGKII) requires its binding to one of the members of the PDZ motif Na^+^/H^+^ exchanger regulatory factor family (NHERF1 and NHERF2) (Weinman et al., 2001; Cha et al., 2005). NHERF1, also known as NHERF or EBP50, and NHERF2, originally termed E3KARP (NHE3 kinase A regulatory protein; (Lamprecht et al., 1998; Yun et al., 1998)) bind to the C-terminus of NHE3 in order to tie it up to the actin cytoskeleton. They each contain two homologous PDZ domains (PDZ1 and PDZ2). Attached to PDZ2 is an ERM (ezrin-radixin-moesin) binding domain by which ezrin links both NHERF1 and NHERF2 to the actin cytoskeleton (Donowitz et al., 2005). In addition, ezrin binds PKAII while cGKII binds to PDZ2, which could explain an additive effect of cAMP and cGMP in inhibiting NHE3 (Donowitz et al., 2005; Donowitz and Li, 2007). Notwithstanding that NHE3 can associate with the actin cytoskeleton by binding to ezrin directly or indirectly *via* NHERF1 or NHERF2 (Cha and Donowitz, 2008), the purpose of this protein complex scaffolding is to position PKAII or cGKII in close proximity to NHE3 in such a way that they can phosphorylate NHE3 and thus regulate its activity and presence in the plasma membrane (Dransfield et al., 1997; Yun et al., 1998; Cha et al., 2005). According to this, knocking down NHERF2 in mice leads to a relocation of NHE3 from submembranous structures into the microvillar membranes of small intestinal epithelial cells, particularly in the distal ileum. This increase in the microvillar location of NHE3 is accompanied by a higher fluid absorptive rate in the ileum of NHERF2 knockout compared to wild type mice, albeit both NHE3 mRNA expression and the acid-activated NHE3 activity, stimulated by an ammonium prepulse-induced intracellular acidification, remain unaffected (Chen et al., 2010). Furthermore, while NHERF2 does mediate the inhibitory effects of cGMP and elevated intracellular Ca^2+^ levels on NHE3 activity in the mouse ileum, it is not required for cAMP-dependent NHE3 inhibition (Chen et al., 2010). In contrast to NHERF2 the IP_3_ receptor-binding protein released with IP_3_ (IRBIT) stimulates NHE3 activity in response to Ca^2+^. IRBIT enhances CaM/CaMKII (Calmodulin/CaM-dependent kinase II)-dependent exocytotic NHE3 trafficking to the plasma membrane (He et al., 2008) and then retains NHE3 in the intestinal brush border membrane by forming a stable protein complex with NHE3, NHERF1 and ezrin (He et al., 2015). In diabetic mice, this macrocomplex falls apart which leads to a loss of NHE3 from the brush border membrane accompanied by diabetic diarrhea. Insulin can initiate the reassembly of the macrocomplex *via* stimulation of PI3K and PKC. PKC phosphorylates and activates ezrin and PKD2 allowing ezrin to interact with NHERF1 and PKD2 to phosphorylate IRBIT. Similar to insulin, orally administered lysophosphatidic acid (LPA) can correct NHE3 localization to the plasma membrane, but interestingly without the need for IRBIT (He et al., 2015). NHERF2, however, is needed for LPA-stimulated, lysophosphatidic acid receptor 5 (LPA_5_)-mediated incorporation of NHE3 into the membranes of the mouse intestinal brush border and a concomitant increase in fluid absorption. Of note, LPA can antagonize the reduced fluid absorption caused by TNFα or by cholera toxin (Lin et al., 2010), which implies its utility as a potential antidiarrheal therapeutic, particularly because it additionally blocks the activation of CFTR-mediated Cl^−^ secretion (Thompson et al., 2018; Tigyi et al., 2019). In the mouse intestine, NHERF1, unlike NHERF2, is not at all required for NHE3 regulation by cyclic nucleotides; however, it does have delicate, segment-specific effects on NHE3 membrane abundance without affecting its mRNA levels, on NHE3 activity, and intestinal salt absorption. NHERF1-deficiency causes a reduction in jejunal fluid absorption, and leads to an attenuated Na^+^ absorption in isolated jejunal and colonic, but not ileal, mucosa. It should be stressed that in these NHERF1 deficient mice, the cAMP-mediated inhibition of fluid and Na^+^ absorption remains unaffected, which points to a third player: the adapter protein NHERF3, also known as PDZK1, which binds to both NHE3 and NHERF1. Knocking down both NHERF1 and NHERF3 causes a complete loss of cAMP-mediated NHE3 inhibition (Broere et al., 2009). A study on interleukin 10 (IL-10)-deficient mice supports the importance of NHERF3 for NHE3 activity including regular intestinal salt and water absorption. These IL-10-deficient mice develop chronic colitis characterized by infiltration of T-lymphocytes and macrophages into the intestinal mucosa accompanied by high levels of the proinflammatory cytokines IL-1β and TNFα, and they excrete pasty stool, the latter indicating diarrheal disease. Although NHE3 expression at mRNA and protein level or its abundance and localization in the apical membrane remain unaffected in these mice, its transport rate is significantly decreased due to 1) an interference with proinflammatory cytokines and 2) a concomitant downregulation of NHERF3 (Lenzen et al., 2012). Most powerful and direct evidence for the causal link between an inflammation-induced loss of NHERF3 and NHE3 dysfunction is provided straight by biopsies from the colon of patients with ulcerative colitis and from inflamed ileal and colonic mouse mucosa. In the inflamed human and mouse intestinal tissues, as compared to healthy control samples, NHERF3 expression is strongly reduced at both mRNA and protein level whereas NHE3 and NHERF1 expression remain unaltered. In addition, NHE3 is properly located in the brush border. The activity of NHE3, however, is significantly lower according to the missing NHERF3 (Yeruva et al., 2015). The inhibition of NHE3 by *Escherichia coli* heat-stable enterotoxin (ST) involves GCC and requires NHERF3-NHERF2 heterodimerization at some point (Yang et al., 2014; Avula et al., 2018). The ST-induced signaling by GCC entails different signaling pathways in different species, also in men and mice, which needs to be taken into consideration in the course of the development of drug therapy (Chen et al., 2019).

Adapter proteins such as NHERFs hold the possibility of interacting with more than one membrane protein. The Cl^−^/HCO_3_^−^ exchanger (SLC26A3), also known as DRA (downregulated in adenoma), cooperates with NHE3 in order to achieve salt absorption by electroneutral ion transport across the apical membrane (Walker et al., 2008). DRA may bind to one of the two PDZ domains of NHERF2 while NHE3 binds to the second one, resulting in a controllable structural link between the functionally coupled NHE3 and DRA (Lamprecht et al., 2002). Another transporter shown to potentially bind to the two PDZ domains of NHERF2 is the putative anion transporter-1 (Pat-1; SLC26A6) (Lohi et al., 2003) that is expressed at significant levels in the epithelium of mouse duodenum where it contributes to basal Cl^−^/HCO_3_^−^ and SO_4_^2−^/HCO_3_^−^ exchange across the apical membrane (Simpson et al., 2007; Walker et al., 2011). A functional coupling between PAT-1 and NHE3 has been suggested to mediate Na^+^HCO_3_^−^ absorption in the mouse jejunum, whereas DRA is predominantly involved in Cl^−^ absorption (Xia et al., 2014).

Apart from NHE3, DRA, and PAT-1, also CFTR can bind to PDZ domains of NHERF2 (Lamprecht and Seidler, 2006). Not only is CFTR required for the PKA-dependent inhibition of Na^+^ absorption driven by NHE3 (Clarke and Harline, 1996; Ahn et al., 2001), but there is a reciprocal interaction between CFTR and NHE3 in terms of PKA-dependent regulation (Bagorda et al., 2002). Although NHE3 and CFTR are thought to regulate each other possibly *via* a common regulatory scaffold protein being part of the protein complex held together by NHERF2, the molecular mechanism of this reciprocal interaction has not been fully understood down to the present day.

### Lipid Rafts and Membrane Curvature Modulate NHE3 Activity

The role of NHERF2 and NHERF3 in NHE3 regulation has been discussed also in the context of lipid rafts. NHERF2 is most raft-associated whereas NHERF3 is entirely non-raft associated so that the differential association of NHERFs with the raft-associated and the non-raft fraction of NHE3 in the brush border membrane may be one reason for the differential and signal specific NHE3 regulation by the different NHERFs (Sultan et al., 2013). Replacement of serine719 by a non-phosphorylatable alanine results in 1) a reduced expression of NHE3-S719A in lipid rafts concomitant with an increased mobile fraction in the brush border, 2) a decreased binding to multiple proteins that normally bind along the NHE3 intracellular terminus, and 3) a decreased transport rate. In addition, not only NHE3-NHERF2 but also NHERF2-NHERF3 binding is considerably decreased, the latter indicating that NHERF2-NHERF3 heterodimerization requires the presence of NHE3 while at the same time the heterodimer serves as a switch to determine whether or not NHE3 regulation is lipid raft dependent (Sarker et al., 2017). Phosphorylation of serine719 by casein kinase two or serine663 by RSK2, respectively, can be induced by LPA *via* LPA5 signaling and eventually leads to an increase in basal NHE3 activity (Sarker et al., 2008; No et al., 2015).

Apart from phosphorylation, scaffolding and trafficking, there is yet another level of acute NHE3 regulation: its activation kinetics can be modulated by the curvature of the plasma membrane. A positive deformation of the plasma membrane by cell swelling upon exposure to hypoosmolar solutions or by changes in its phospholipid composition drives the transition from a first inactive conformation of NHE3 to a second one, which then can respond more rapidly to physiological stimuli, probably depending on the cytosolic H^+^ concentration (Alexander et al., 2007).

### Exploiting NHE3 as a Therapeutic Target

Not despite but because of its complexity, the regulatory mechanisms of NHE3 can be taken advantage of, for instance, in cystic fibrosis (CF). CF patients suffer from a loss of CFTR activity, which causes dehydration of intestinal contents and eventually life-threatening obstructions. CFTR knockout mice lacking one or both copies of the NHE3 gene show an increase in the fluidity of their intestinal content and less obstruction, because more of the osmotically and electrostatically active Na^+^ remains in the gut lumen (Bradford et al., 2009). This condition of no or reduced NHE3 activity can be reached also by inhibiting one or more of the various parameters regulating NHE3, or by blocking NHE3 itself. Tenapanor, available as tenapanor hydrochloride (IBSRELA), inhibits NHE3, is orally available and only minimally absorbed in the gastrointestinal tract. As long as administered orally, it acts locally but not systemically, therefore, has little toxicity and hardly side effects. It has been successfully applied to treat patients suffering from irritable bowel syndrome with constipation (Zielińska et al., 2015; Sinagra et al., 2020; Chey et al., 2021). Also in CF patients, tenapanor may be used to ameliorate constipation and reduce obstructive episodes. In CFTR null and F508 del mutant mouse intestine, direct NHE3 inhibition by tenapanor reduces the fluid absorptive rate and increases alkaline output (Tan et al., 2021). As implied above, interfering with the molecular mechanisms regulating NHE3 activity can result in beneficial effects as well. Thus, the GCC agonist linaclotide can be used to treat potentially both patients presenting with irritable bowel syndrome with constipation and CF patients (Chey et al., 2012; Tan et al., 2021). In addition, the PGE1 (prostaglandin E1) analogue lubiprostone, which can induce signaling cascades mediated by prostaglandin receptors EP1 and EP4 and primarily stimulates Cl^−^ secretion *via* CFTR while at the same time suppressing the incorporation of NHE3 into the brush border membrane (Jakab et al., 2012), may be used to attenuate constipation in CF patients (Tan et al., 2021).

### Conclusion

Although fairly complex, the sophisticated regulation of NHE3 at every level, from transcription and translation to recycling between brush border and intracellular membranes to modulation of its activity not only by physical or physiological interaction with other transporters and channels, holds enormous potential for therapeutic exploitation. Theoretically, each regulatory step might serve as a therapeutic target. Patients with diarrheal diseases may profit from NHE3 stimulation whereas NHE3 inhibition helps to counteract constipation. On the other hand, these extensive NHE3 regulatory interdependencies require a precise and thorough control of all possibly occurring side effects, which include changes in diuresis and blood pressure because NHE3 plays a major role in renal Na^+^ and H_2_O retention as well (Li et al., 2019).

## NHE4 in the Gastrointestinal Epithelium

NHE4 was first cloned from rat brain, heart, kidney, stomach, and spleen cDNA libraries, which were screened for yet undiscovered NHE isoforms using an NHE-1 cDNA probe under low stringency hybridization conditions (Orlowski et al., 1992). When the NHE4 probe was used for Northern hybridization, transcripts were found to be most abundant in stomach, followed by small and large intestine, and only weak bands in kidney, brain and uterus. The Northern blot results only partially reflected the results later obtained with more specific cDNA probes, in which the strong gastric expression, as well as the expression in brain, kidney and uterus was confirmed and later on were connected to a function of NHE4 in these organs (Bookstein et al., 1996; Wang et al., 2003; Bourgeois et al., 2010; Sakuta et al., 2020) whereas no amplification products were found in rat intestine (Bookstein et al., 1997). Nevertheless, colonic crypt or colonic cell line NHE4 expression and function have been reported by some (Beltrán et al., 2008; Arena et al., 2012), and not by others (Bookstein et al., 1997; Janecki et al., 1999; Magro et al., 2005).

Because our group had studied and found differences in the properties and regulation of Na^+^/H^+^ exchangers in the different gastric epithelial cell types (Seidler et al., 1992), we were eager to identify the cell-type specific NHE isoform expression in rat and rabbit gastric epithelial cells (Rossmann et al., 2001). Indeed, while also expressing NHE1 and NHE2, rabbit and rat parietal cells displayed the highest NHE4 expression levels. Making use of the fact that NHE4 is insensitive to all NHE inhibitors except high dimethyl-amiloride concentrations, NHE4 was found to be the parietal cell NHE isoform most strongly stimulated by cAMP stimulation, while Ca^2+^-dependent agonists stimulated NHE1 activity (Bachmann et al., 1998). Because muscarinic stimulation elicits only a small acid secretory response in isolated parietal cells, whereas cAMP-dependent stimulation evokes a strong secretory response, it was speculated that the NHE4 activation was secondary to secretagogue-associated cell volume changes. Indeed, parietal cell NHE4 appeared to be not activated by low pH_i_, but by hyperosmolarity. Subsequent experiments fluorometrically assessed cytoplasmic volume changes in cultured parietal cells during stimulation of acid formation, and could find evidence for shrinkage-mediated NHE4 activation and for supporting a role of NHE4 in addition to NHE1 in parietal cell volume regulation (Sonnentag et al., 2000; Bachmann et al., 2007).

The generation of *nhe4* ^−/−^ mice confirmed the involvement of NHE4 in stimulated acid secretion, because the *nhe4* ^−/−^ mice had a hypochlorhydric stomach content and displayed reduced numbers of structurally abnormal parietal cells (Gawenis et al., 2005; Miller et al., 2010). It is not clear why NHE4 activation during acid secretion is essential and in particular, why the *nhe4* ^−/−^ parietal cells develop such a marked morphological alteration. Possibly, the strong intracellular generation and basolateral extrusion of base, the so called “alkaline tide”, maintains NHE1 in quiescence, and the volume regulation required for RVI needs to be performed by NHE4 (in conjunction with the Cl^−^/HCO_3_^−^ exchanging anion-exchanger AE2). The other GI epithelia in which NHE4 expression has been reported have not been investigated functionally in the *nhe4* ^−/−^ mouse; no morphological abnormalities were detected.

Can we get further elucidation on parietal cell NHE4 function and regulation by looking at other epithelia? NHE4 is also expressed in the basolateral membrane of the cells of the medullary thick ascending limb of Henle’s loop (MTALH), together with NHE1. Similar to the situation in parietal cells, NHE1 performs the major part of pH_i_-recovery from an intracellular acid load in the MTALH cells (Bourgeois et al., 2010). Nevertheless, *nhe4* ^−/−^ mice display a defect in ammonia absorption in the MTALH, and cannot secrete an excess acid load by upregulating renal ammonium secretion. The idea is that ammonium ions (NH_4_^+^) enter *via* the luminal membrane, transported by a variety of transport systems. In the cell, NH_4_^+^ generates NH_3_ which leaves the basolateral membrane *via* diffusion, and H^+^ which is in part neutralized by NBCn1-imported HCO_3_^−^ and in part exported as NH_4_^+^ or H^+^ *via* NHE4 (Blanchard et al., 1998). Why NHE4 and not NHE1 is not clear, but possibly related to the high extracellular sodium concentration in the interstitium of the renal medulla (or specific properties of the NHE4 protein itself). The activating effect of extracellular Na^+^, but not of hyperosmolarity per se, on NHE4-mediated Na^+^/H^+^ exchange was recently demonstrated in cells from the *organum vasculosum* of the *lamina terminalis* (OVLT) in the brain (Sakuta et al., 2020). We know little about the extracellular milieu at the parietal cell’s basolateral membrane, but it is known that acid secretion is associated with a large initial luminal K^+^ loss by the parietal cell and membrane depolarization, which needs to be compensated by the Na^+^/K^+^ ATPase, because the acid-secreting parietal cells are not dependent on NKCC cotransport (McDaniel and Lytle, 1999; Miller et al., 2002; McDaniel et al., 2005). Therefore, it is feasible that the Na^+^ concentration at the parietal cell’s basolateral side may rise quickly after the onset of acid secretion. Further studies are necessary to unravel the complexities of gastric epithelial ion transport.

## NHE8 in the Gastrointestinal Tract

### Cloning, Regulation and Kinetic Properties of NHE8

Investigation of the NHE–mediated Na^+^ absorption in the renal proximal tubule of NHE3 or NHE3/NHE2 null mice revealed the presence of an EIPA-sensitive Na^+^-dependent acid extrusion across the apical membrane that was not attributable to the NHE isoforms known at that time (Choi et al., 2000). The new NHE isoform was soon after cloned from a mouse kidney cDNA library and identified as NHE8 (Goyal et al., 2003). Led by this discovery, Xu et al. cloned the intestinal NHE8 (Xu et al., 2005).

NHE8 expression in the intestine is age dependent, with the highest expression in young animals (Xu et al., 2005; Xu et al., 2008; Fiori et al., 2009; Xu et al., 2012). Several studies have shown that the binding of the Sp3 transcriptional factor to the NHE8 basal promoter is crucial for the transcriptional regulation of NHE8 (Xu et al., 2009; Xu et al., 2010a; Xu H. et al, 2015). TNF-α inhibits NHE8 expression in Caco2 cells by reducing Sp3 interaction at the NHE8 promoter (Xu et al., 2009). Similarly, EGF treatment can downregulate NHE8 mRNA and protein expression in suckling rats and Caco2 cells by hindering Sp3 transcriptional factor interaction with the NHE8 basal promoter (Xu et al., 2010a). In contrast, short chain fatty acids seem to have a stimulating effect on NHE8 expression, since treatment with butyrate increased NHE8 mRNA and protein abundance in Caco2 cells *via* binding of Sp3 to the NHE8 basal promoter (Xu H. et al, 2015). Somatostatin (a peptide produced in the GI tract, with pro-absorptive and anti-secretory properties), was shown to have a stimulating effect on NHE8 expression and activity in the mouse intestine and Caco2 cells *via* p38 mitogen-activated protein kinase (MAPK) activation (Wang et al., 2011). NHE8 expression can be transcriptionally regulated by glucocorticoids also, as it was shown that methylprednisolone administration reduced NHE8 mRNA expression in the rat small intestine and Caco2 cells by binding of the Pax5 transcription factor to the NHE8 gene promoter (Xu et al., 2010b).

Metabolic acidosis caused increased NHE8 protein expression and activity in the brush-border membrane of renal proximal tubule (Twombley et al., 2010). In NRK renal cells, where NHE8 is the only NHE isoform expressed in the apical membrane, NHE8 protein expression and Na^+^/H^+^ exchange activity was significantly increased when cells were cultured in acidic media (Joseph et al., 2012). The effect was not transcriptionally regulated as the protein abundance was not affected by actinomycin D or cycloheximide, nor was the NHE8 mRNA expression affected by the acidic medium (Joseph et al., 2012). We investigated the role of acidosis on NHE8 expression as well, triggered by the finding that NHE2 knock-down Caco2BBe cells with chronically reduced pH_i_ show a significant increase of NHE8 mRNA expression (Yu et al., 2019). By manipulating the pH_i_ of Caco2BBe cells *via* changing the pH_e_ of the medium (pH_e_ 6.8, 7.3, or 7.8), we showed that acidification of the pH_i_ stimulated, whereas alkalization decreased, NHE8 mRNA expression compared to cells with physiologic pH_i_ ∼7.3 (Zhou et al., 2021), thus uncovering a transcriptional mode of NHE8 regulation in Caco2BBe cells. The difference in the two studies is that the first one represents a short (acute) treatment with acidic media (24 h) (Joseph et al., 2012), while in the second study, the cells were exposed chronically to acidic medium (14 days) (Zhou et al., 2021).

Goyal et al. detected NHE8 using a rabbit polyclonal antibody raised against the COOH-terminal hydrophilic tail of human NHE8, and identified a 85 kDa protein, unlike the 64 kDa predicted from the length of the open reading frame. The discrepancy was explained by possible glycosylation during posttranslational modification, as the existence of four N-glycosylation sites has been predicted (Goyal et al., 2003). In intestinal tissue and lysates from Caco2 cells, the group of Ghishan [using a self-made antibody (Xu et al., 2005)] and our group [using heterogeneously expressed NHE8 with a Flag tag (Zhou et al., 2021)] detected NHE8 with a molecular mass of ∼64 kDa. Of note, most of the commercially available NHE8 antibodies also detect a protein with 85 kDa, and were shown to be unspecific in our hands.

Unlike for NHE1, NHE2 and NHE3, the half-life of NHE8 is still not determined, but its kinetic properties have been characterized in a study using NHE deficient PS120 cells transfected with rat NHE8 cDNA. As shown in Table 2, the study determined that NHE8 had a K_m_ [pH_i_] = pH 6.5, and a K_m_ [Na^+^] = 23 mM (Xu et al., 2008), which brings it closer to the kinetic properties of NHE3, but not NHE1 and NHE2 (Orlowski, 1993; Yu et al., 1993).

### Pharmacological Inhibition of NHE8

NHE8 was discovered after detecting residual NHE activity in *nhe3* ^*−/−*^ mice, that was sensitive to EIPA (Choi et al., 2000). Since then the sensitivity of NHE8 to EIPA has been shown in several studies (Fiori et al., 2009; Kang’ethe et al., 2007; Wiebe et al., 2019; Zhang et al., 2007). In PS120 cells expressing rat NHE8, HOE694 (10 μmol/L) significantly reduced the activity of NHE8 (Xu et al., 2008). S3226, an NHE3-specific inhibitor (Schwark et al., 1998), could also inhibit NHE8 activity at a concentration of 80 μM (Xu et al., 2008). In our recent study, using NHE-deficient PS120 cells transfected with human and rat NHE8 cDNA, we have shown that tenapanor [another NHE3-specific inhibitor (Spencer et al., 2014)] has no inhibitory effect on NHE8 at the concentration used for NHE3 inhibition (Zhou et al., 2021). Using the same model, we addressed NHE8 sensitivity to HOE642 an inhibitor known to inhibit NHE2 and NHE1, but not NHE3 (Scholz et al., 1995; Yu et al., 2019)] and found that NHE8 is inhibited by 3 µM HOE642 (Zhou et al., 2021). The same concentration of HOE642 inhibited NHE1 activity in the basolateral membrane (Paehler Vor der Nolte et al., 2017; Yu et al., 2019; Zhou et al., 2021). With this information in hand, we were able to separate the activity of NHE1, NHE2, NHE3 and NHE8 expressed endogenously in a Caco2BBe cell line (Yu et al., 2019; Zhou et al., 2021), which would not have been possible using another inhibitor such as HOE694 or S3226, due to the overlapping inhibitory profiles for some of the NHE isoforms.

### Expression of NHE8 in the Gastrointestinal Tract

NHE8 was firstly described in the proximal tubule of kidneys as an active ion transporter in the apical membrane (Goyal et al., 2003) and was later shown to be located in both microvillar surface membranes and the coated pit regions in the epithelial cells of renal proximal tubules (Goyal et al., 2005). The NHE8 mRNA expression pattern was analyzed by Northern blot analysis using human tissue RNA blots which showed wide distribution of NHE8 expression in almost all tissues, especially heart, lung, skeletal muscle, intestine, kidney, liver, and placenta (Xu et al., 2005). In the gastrointestinal tract, NHE8 expression displays segmental differences, with higher NHE8 expression in the stomach, duodenum, and ascending colon in humans, while in mice, higher NHE8 expression was observed in the jejunum, ileum, and colon (Xu et al., 2008). The distribution of NHE8 along the gastrointestinal tract is shown in Figures 1–3.

Since its identification, the exact (sub)cellular localization of NHE8 has been a big enigma. Although, NHE8 protein was detected in brush border membrane protein preparation of renal cells (Goyal et al., 2003), other studies showed conflicting results. Namely, that NHE8 was localized to the mid- and trans-Golgi in COS-7 cells with heterologous expression of NHE8. Overexpression of NHE8 increased the pH of the trans-Golgi in these cells by ΔpH∼0.7 to near cytosolic pH, indicating that NHE8 acts as an Na^+^/H^+^ exchanger in this organelle (Nakamura et al., 2005). NHE8 was also detected in the ER, Golgi and intracellular vesicles of the retinal pigment epithelium (Xia et al., 2015). However, in the renal cell line NRK (Joseph et al., 2012; Wiebe et al., 2019), the intestinal epithelial cell line Caco2 (Xu et al., 2009; Xu et al., 2010a; Xu H. et al, 2015) and HT29 (Xu et al., 2016; Xu et al., 2019), as well as the mammalian intestinal epithelium (Xu et al., 2016; Lei et al., 2018; Xu et al., 2019) NHE8 is localized in the apical membrane. Nevertheless, a close look at all the published immunofluorescence images obtained with NHE8 antibodies shows a small NHE8 fraction in the cytoplasm, indicating that the occurrence of NHE8 may not necessarily be restricted/limited to membranes. Since most of the mentioned studies used models where NHE8 is overexpressed, utilization of knock-down experiments were required to confirm that endogenous NHE8 is involved in the organellar or cytosolic pH regulation. To this end, we have recently generated an NHE8 knock-down Caco2BBe cell line, in which NHE8 mRNA expression was decreased by ∼ 60% (Zhou et al., 2021). Using pH fluorometry and a double perfusion chamber to separate the basolateral and apical NHE activity, we were able to show that downregulation of NHE8 expression caused reduced net apical NHE activity in this cell line (Zhou et al., 2021). Furthermore, the pH_i_ of the Caco2BBe cells lacking NHE8 was significantly more acidic compared to the control, thus demonstrating that NHE8 is an active Na^+^/H^+^ transporter in the apical membrane of Caco2BBe cells. The plasma membrane residence of the NHE8 was confirmed by heterologous expression of NHE8 tagged with Flag, which also showed fractions of intracellularly located protein (Zhou et al., 2021). It is possible that NHE8 resides in intracellular vesicles and when they fuse with the apical membrane it becomes an active brush border transporter like it is described for other transporters [for example the gastric proton pump and NHE3 (Forte and Yao, 1996)]. It may also have an active role in regulating vesicle pH and/or volume in the intracellular vesicles [like in the case of ClC_3_ and V-type H^+^ ATPase (Thévenod, 2002)]. Therefore, the reported discrepancies between subcellular or apical membrane localization of NHE8 in epithelial cells may represent variations in protein trafficking, differences between cell types, or technical differences between different groups. However, this question remains open and awaits development of a more specific NHE8 antibody and studies on NHE8 activity in the compartments where it is expressed.

### NHE8 Activity in the Gastrointestinal Tract

In the stomach, NHE8 is detected in the apical membrane of the surface mucus cells (Xu et al., 2013), and it was reported that *nhe8* ^*−/−*^ mice have a reduced gastric mucosal surface pH and a higher incidence of developing gastric ulcer (Xu et al., 2013). The finding indicated a possible defect in the bicarbonate secretion of the *nhe8* ^*−/−*^ mice. This was confirmed by the reduced expression of DRA in the affected cells (Xu et al., 2013). The study revealed a possible role of NHE8 in the stomach by participating in gastric mucosal protection.

In the intestine, NHE8 is also expressed in the brush-border membrane (Xu et al., 2005). In the intestine of young mice, NHE8 mRNA expression is even more prominent than that of NHE2 and NHE3, suggesting an important role of NHE8 during early development (Xu et al., 2011). Due to its high expression in young mice before weaning, NHE8 was proposed as a compensatory mechanism for NHE2 and NHE3 loss in *nhe2* ^*−/−*^ */nhe3* ^*−/−*^ mice (Xu et al., 2011). The insinuation was based on the higher survival rate of the young female mice compared with the male double-knockout mice, coinciding with higher NHE8 expression in the female mice until the age of 6 weeks (Xu et al., 2011). However, there is no functional evidence that NHE8 contributes to the Na^+^ absorption in young mice and would thus compensate for the loss of NHE3. The *nhe8* ^*−/−*^ mice have no obvious intestinal phenotype, show no defect in Na^+^-absorption, have normal serum Na^+^ levels and no signs of diarrhea (Xu et al., 2012). Interestingly, increased expression of both NHE2 and NHE3 is detected in the small intestine, but not in the colon of the *nhe8* ^*−/−*^ mice. In the renal proximal tubule NHE3 and NHE8 reciprocally compensate for each other: NHE8 expression in the BBM of this epithelium was increased in *nhe3* ^*−/−*^ mice, and vice versa in the *nhe8* ^*−/−*^ renal proximal tubule elevated NHE3 expression was reported (Baum et al., 2012). In the Caco2BBe cells, NHE8 mRNA expression and activity was increased in the apical membrane of NHE2-knockdown cells, thus rescuing the loss of NHE2 as the net NHE apical activity was not different between NHE2-knockdown and control Caco2BBe cells (Yu et al., 2019; Zhou et al., 2021). Conversely, however, NHE2 did not compensate for the loss of NHE8, since NHE8-knockdown cells displayed significantly reduced NHE2 mRNA expression (Zhou et al., 2021). A possible explanation could be that NHE8 was activated by the acidic intracellular pH_i_ in the NHE2-knockdown Caco2 cells, since NHE8 mRNA expression was stimulated by acidosis, whereas the NHE2 expression was not affected by the intracellular pH_i_ (Zhou et al., 2021; Nikolovska et al., 2022).

In the colon of *nhe8* ^*−/−*^ mice, significant acidification of the mucosal surface was observed, by ∼0.14 pH units in the proximal colon and by ∼0.47 pH units in the distal colon, possibly due to a reduced expression of Slc26a3 (DRA). Furthermore, the secretory lineage differentiation was strongly impacted in the *nhe8* ^*−/−*^ colon with significant reduction of the goblet cells number accompanied by decreased Muc2 mRNA expression (Xu et al., 2012). Since lineage differentiation was also affected in the colon of *nhe2* ^*−/−*^ mice, with reduced absorptive, but increased secretory cell differentiation (Nikolovska et al., 2022), this raised the question whether both NHE2 and NHE8 are activated during initiation of cell lineage differentiation, with NHE2 predominance in absorptive cells, and NHE8 predominance in secretory cells. NHE8 expression is more abundant in goblet cells than in enterocytes, since NHE8 mRNA expression was significantly higher in HT29-MTX cells (a mucus-producing HT29 subclone) compared to Caco2BBe cells (Xu et al., 2016; Zhou et al., 2021). Furthermore, the observed significant increase in NHE8 mRNA expression in differentiated versus undifferentiated HT29 cells points to a special role of NHE8 in goblet cell differentiation. In this case, the potential subcellular localization of NHE8 is even favorable since it will allow the presence of an active Na^+^/H^+^ exchanger in the mucus granules of the goblet cell that could mediate the pH fluctuation necessary for mucin exocytosis (Chin et al., 2002) or assist membrane trafficking of other proteins, such as Slc26a3 (DRA) (Lissner et al., 2010). The physiological importance of colonic NHE8 expression is highlighted by the impaired mucosal protection seen in *nhe8* ^−/−^ colon due to altered mucus layer formation, resulting in increased bacterial adhesion, with a significant increase in Bacteroidetes, *Lactobacillus*, and Firmicutes bacteria and in segmented filament bacteria (Liu et al., 2013). In an attempt to rescue the mucosal phenotype observed in *nhe8* ^*−/−*^ colon, the group used fecal microbiota transplantation (FMT), feeding the mice with the probiotic VSL#3 or administration of sodium butyrate *via* enema, however none of the approaches was able to restore mucin production and the dysbiosis caused by the absence of NHE8 (Bernardazzi et al., 2020).

Literature data on the physiological role of intestinal NHE8 is scarce and limited to the same group that cloned the intestinal NHE8 and generated the *nhe8* ^*−/−*^ mice (Xu et al., 2005; Xu et al., 2012). Although their contribution to revealing the functional characteristics of NHE8 is enormous, further investigation is necessary to clarify the subcellular localization and activity of NHE8 in different segments of the gastrointestinal tract.

### NHE8 in Pathophysiology of the Gastrointestinal Tract

Mucosal barrier integrity is one of the most important features of the gastrointestinal tract and its impairment is considered an etiologic factor in the pathogenesis of IBD (Pastorelli et al., 2013). The loss of the mucus barrier in epithelial cells lacking NHE8, implies that NHE8 expression and function might be affected in IBD. Indeed, in ulcerative colitis (UC) patients, NHE8 expression was reduced (Li et al., 2016). Impaired expression of NHE8 was detected in animal colitis models as well (Xu et al., 2009; Li et al., 2016). Furthermore, manifestation of spontaneous colitis with microbial dysbiosis, increased epithelial cell proliferation, and high susceptibility to DSS-induced colitis are reported in mice lacking NHE8 (Wang et al., 2015). It has been recently shown that NHE8 is an important susceptibility gene for a reduced β-diversity in the human microbiome (Rühlemann et al., 2018).

A recent publication opened the question of a possible implication of NHE8 in colonic tumorigenesis (Xu et al., 2019). The study demonstrated that NHE8 expression is strongly reduced in colorectal cancers and *nhe8* ^*−/−*^ mice show a higher tendency to develop tumors in the azoxymethane/dextran sodium sulfate colon cancer model. Furthermore, HT29^NHE8KO^ cells showed increased propensity for colony formation and formed larger tumors when injected in NSG mice, compared to HT29^NHE8 wild type^ cells (Xu et al., 2019). The increased proliferation in cells lacking NHE8 was confirmed by increased Lgr5 expression and increased Wnt/β-catenin activation in the colon, in HT29-derived tumors, and in colonoids (Xu et al., 2019). Although NHE8-deficient Caco2BBe cells had a higher proliferation rate as well, they showed no alteration in the Wnt/β-catenin signaling pathway, but instead an activation of the EGF signaling pathway (Zhou et al., 2021).

Even though the NHE8 deficiency is demonstrated in ulcerative colitis and tumorogenesis, the exact mechanism of interaction with the procancerogenic growth factors or the inflammatory cytokines is not very well researched and should be addressed in the future.

## Conclusion and Outlook

The five NHE isoforms found in the plasma membranes of gastrointestinal epithelial cells, NHE1-4 and NHE8, act in concert to ensure both barrier integrity by the continuous renewal of the mucosa based on cell proliferation, differentiation and migration, and basic epithelial functions such as fluid and electrolyte transport.

The basolaterally located NHE1 is in charge of housekeeping functions including pH_i_ homeostasis and volume regulation. It contributes to wound healing and regeneration by being involved in cell proliferation and migration. NHE1 also acts as a stabilizing structural component as it physically interacts with a number of proteins, which links it to the cortical actin cytoskeleton. NHE1-mediated H^+^ extrusion plays an essential role in the supply of intracellular HCO_3_^−^ during stimulated HCO_3_^−^ secretion in the duodenal mucosa.

The apically located NHE2 most likely contributes to the differentiation of 1) parietal cells during epithelial restitution and 2) progenitor cells in the transit amplifying progenitor zone of the colonic crypt. Thus, NHE2 deficiency shifts the differentiation program of colonic epithelial cells from the absorptive towards the secretory lineage. Although the Wnt/Notch signaling pathways in NHE2 deficient colonocytes are clearly altered, the question of how the decreased cytosolic pH_i_ observed in colonic progenitor cells acts on their differentiation program needs to remain unanswered at this point.

NHE3, located also apically, orchestrates intestinal salt and water absorption in close coordination with CFTR and DRA. Its activity in both the intestine and the kidney makes NHE3 one of the major players in systemic electrolyte, volume and blood pressure regulation. Based on its physiological importance combined with a highly complex, multifaceted regulation, NHE3 including its molecular regulators hold enormous potential as therapeutic targets to treat diarrheal diseases as well as constipation, particularly in CF patients.

NHE4 is found in the basolateral membrane of parietal cells where its activity is required in response to cytoplasmic volume changes during stimulated acid secretion. In cultured parietal cells, its activity can be triggered by shrinkage, which points to its function as cell volume regulator in these cells in addition to NHE1.

NHE8 has the task of contributing to mucosal protection in the stomach and the intestine. In the colon, NHE8 deficiency leads to an altered mucus layer formation resulting in an undesirably increased adhesion of unwanted bacteria. Consistent with its role in mucosal protection NHE8 predominates in secretory cells whereas NHE2 predominates in absorptive cells. This may imply a role of NHE8 in goblet cell differentiation.

With the objective of finding out more about the mission of NHEs in the development and differentiation pattern of gastrointestinal cells as well as their contribution to microbial resistance by controlling the mucosal barrier, promising methodological progress has been made recently. The generation and development of epithelium-derived organoids from all gastrointestinal organs including the preservation of their specific function(s) opens up the possibility of investigating more deeply the different physiological roles of NHEs and their pharmacology under precisely defined conditions.
